# Comprehensive Analysis Reveals USP45 as a Novel Putative Oncogene in Pan-Cancer

**DOI:** 10.3389/fmolb.2022.886904

**Published:** 2022-06-28

**Authors:** Kai Li, Qian Wang, Hua Bian, Zhiguo Chen, Haifa He, Xulin Zhao, Pengju Gong

**Affiliations:** ^1^ Zhang Zhongjing College of Chinese Medicine, Nanyang Institute of Technology, Nanyang, China; ^2^ Henan Key Laboratory of Zhang Zhongjing Formulae and Herbs for Immunoregulation, Nanyang Institute of Technology, Nanyang, China; ^3^ School of Basic Medical Sciences, Xinxiang Medical University, Xinxiang, China; ^4^ Department of Pathology, Central Hospital of Nanyang City, Nanyang, China; ^5^ Department of Oncology, The First People’s Hospital of Nanyang, Nanyang, China; ^6^ The University of Texas MD Anderson Cancer Center UThealth Graduate School of Biomedical Sciences, Houston, TX, United States

**Keywords:** deubiquitinating enzyme, USP45, pan-cancer analysis, tumor immunotherapy, TCGA (The Cancer Genome Atlas Program)

## Abstract

**Background:** Deubiquitinating enzymes specifically removes ubiquitin molecules from ubiquitin-tagged target proteins, thereby inhibiting the degradation of target proteins and playing an important role in tumor. However, the mechanism of deubiquitinating enzyme USP45 in tumors remains unclear.

**Methods:** Based on the RNA-seq data of tissues and cell lines in The Cancer Genome Atlas (TCGA) database, GTEx and CCLE database, the pan-cancer analysis of USP45 expression and survival outcome were performed using R software and Kaplan-Meier Plotter. The structural variants, gene mutations and gene copy number alteration of USP45 were analyzed using the TCGA Pan-Cancer Atlas Studies dataset in the cBioPortal database. The relationships between USP45 and mRNA methylation, tumor heterogeneity, tumor stemness, and tumor immunity were performed by Sangerbox platform and TIMER2.0 using Pearson correlation analysis. Through the ENCORI database and string database, we constructed the ceRNA regulatory mechanism and protein-protein interaction network for USP45. Based on the RNA-seq data in TCGA and GTEx databases, we also constructed the downstream regulatory network for USP45 using the Limma and ClusterProfiler packages of R software. At last, the protein expression levels of USP45 were detected by immunohistochemistry in tumor tissue microarrays.

**Results:** USP45 is upregulated in most types of tumors and negatively correlated with the overall survival and recurrence-free survival of patient. Furthermore, the structural variation, gene mutations and gene copy number variation of USP45 were identified in different types of tumors. The pan-cancer analysis showed that USP45 was closely related to mRNA methylation, tumor heterogeneity and tumor stemness. In most types of tumors, the expression of USP45 was positively correlated with many immune checkpoint molecules and immune regulators such as PD-L1, while negatively correlated with the infiltration levels of NK cells, Th1 cells, macrophages, and dendritic cells in the tumor microenvironment. Finally, we constructed the ceRNA regulatory network, protein-protein interaction network and downstream regulatory network for USP45 in different types of tumors.

**Conclusion:** Our study firstly explored the putative oncogenic role of USP45 in pan-cancer, and provided insights for further investigation of USP45.

## Introduction

Pan-cancer research has been an important development in recent years, which is helpful to reveal the mechanisms of genes, especially the genes poorly understood, in the development of different tumor types. In this study, we took USP45 as our subject and revealed its important role in the development of different types of tumors through pan-cancer analysis.

USP45 is a member of the USPs (ubiquitin-specific proteases) family (Conte et al., 2018). Over 100 deubiquitinating enzymes (DUBs) have been identified (Tyagi et al., 2021). These DUBs can be divided into six families, including USPs (ubiquitin-specific proteases), UCHs (ubiquitin c-terminal hydrolases), MJDs (proteases containing the Machado-Joshphin region), OUTs (ovarian cancer proteases), MINDYs (motif-interacting with ubiquitin-containing novel DUB family), and JAMMs (JAB1, MPN, MOV34 family). Deubiquitinating enzymes play an important role in the ubiquitin-protease system by cleaving the linkages between the ubiquitin chain and the substrate protein or between the ubiquitinated chains, allowing the ubiquitin molecule to be detached from the protein substrate. Ubiquitination controls the stability of most intracellular proteins, and its abnormalities can lead to a wide range of diseases, including cancer (Lange et al., 2022). The inhibition of deubiquitinating enzymes can lead to selective protein degradation and may affect other “drug-free” targets. Therefore, USPs may be promising drug targets, and several inhibitors of USPs have been developed and have shown promising therapeutic effects. For example, PR619, a broad-spectrum inhibitor of DUBs, can modulate microtubule networks by inhibiting deubiquitinating enzymes, thereby improving neurodegenerative diseases (Seiberlich et al., 2012). The USP7 inhibitor FT671 has a significant anti-tumor effect in a mouse model by upregulating p53 levels (Turnbull et al., 2017). Auranofin, an inhibitor of USP14, is currently in phase 2 clinical trials as an anti-tumor agent (Liu et al., 2019).

At present, the function of the USP45 is mostly unknown. USP45 was found to promote cell migration by catalyzing the deubiquitination of SPDL1 protein and upregulating its protein expression level (Conte et al., 2018). In a zebrafish model, knockdown of USP45 resulted in moderate to severe ocular morphological defects. This phenotype is associated with retinal formation and differentiation, suggesting that USP45 is a key gene causing hereditary retinal dystrophies (Toulis et al., 2016; Yi et al., 2019). USP45 has also been found to be a key regulator of DNA repair, and cells lacking USP45 are particularly sensitive to UV irradiation and DNA interstrand cross-linking, with significantly reduced repair capacity for UV-induced DNA damage. Its mechanism is mainly to catalyze the deubiquitination of ERCC1 and promote its recruitment to DNA damage sites (Perez-Oliva et al., 2015). However, no studies have yet revealed the mechanism of USP45 in tumor development.

In this study, we used bioinformatics to explore the expression levels and mutations of USP45 in different types of tumor and their correlation with patient prognosis, mRNA methylation, tumor heterogeneity, tumor stemness, and tumor immunity. In addition, we also constructed the ceRNA regulatory network, protein-protein interactions network, and downstream regulatory network for USP45. Finally, we verified the expression level of USP45 in 12 types of tumors using immunohistochemistry. In conclusion, our study showed that USP45 acted as a novel putative oncogene and might play an important role in the development of pan-cancer.

## Materials and Methods

### Data Collection and Gene Expression Data Mining

The normalized RNA-Seq data of 33 types of cancers were downloaded from the TCGA database using UCSC Xena (https://xena.ucsc.edu/). Gene profile data of human normal tissues were retrieved from GTEx (https://commonfund.nih.gov/GTEx). Besides, the gene expression data of cell lines was obtained from the CCLE database (https://portals.broadinstitute.org/ccle/about). We further extracted the expression data of the ENSG00000123552 (USP45) gene in each sample based on the filter principle as Solid Tissue Normal, Primary Solid Tumor, Primary Tumor, and Normal Tissue. Then, log2(x + 1) transformation was performed on each expression value. Finally, the cancer type whose sample size was less than three would be eliminated. Wilcoxon Rank Sum and Signed Rank Tests were employed to compare the expression level between two groups, *p* < 0.05 was considered to be statistically significant using R software (version 3.6.4) and results were visualized by the ggplot2 package.

### Survival Outcome Analysis

The prognostic role of USP45 among different types of cancers in the TCGA database was investigated by the online database and survival analysis tool, Kaplan-Meier Plotter (http://kmplot.com/analysis/index.php?p=service&cancer=pancancer_rnaseq). To assess the overall survival (OS) and Recurrence-free survival (RFS) of patients in pan-cancer, patients’ samples were divided into high and low USP45 expression groups by the best cut-off value automatically and were analyzed by using Kaplan-Meier analysis and Log rank-P test (*p* < 0.05 as significant).

We used MethSurv (https://biit.cs.ut.ee/methsurv/) to determine the prognostic value of single CpG methylation of USP45 in pan-cancer. In this analysis, DNA methylation values were represented using beta values (beta values ranging from 0 to 1). Every single methylation of CpG was calculated by the M/(M + U + 100) formulation. M and U are methylated and unmethylated intensity values.

### Genetic Alteration Analysis

The TCGA Pan-Cancer Atlas Studies dataset in the cBioPortal database (https://www.cbioportal.org/) was utilized for genetic alterations evaluation of USP45. The results of the alteration frequency, mutation type, and Copy number alteration (CNA) across all TCGA tumors were visualized in the “Cancer Types Summary” module. Then, the correlations between mRNA expression levels of USP45 and CNA were calculated by using Wilcoxon Rank Sum and Signed Rank Tests for significant difference analysis between two groups, and Kruskal-Wallis test for multi-group difference test.

### USP45-Related Gene Enrichment Analysis

Firstly, the Similar Gene Detection module of GEPIA2 (http://gepia2.cancer-pku.cn/#similar) was used to obtain the top 200 of USP-correlated genes based on the gene expression data of all TCGA tumor samples. Then, Functional enrichment analysis including Gene Ontology (GO) analysis and Pathway enrichment of the 200 genes was performed with the Metascape website (http://metascape.org/gp/index.html#/main/step1).

### Tumor Heterogeneity and Stemness Features Analysis

The correlations between USP45 and tumor heterogeneity, stemness features were analyzed by online tool Sangerbox (http://vip.sangerbox.com/) using Pearson’s correlation method with all types of TCGA tumor samples. Six Stemness Scores were calculated using mRNA and DNA methylation profiles, including RNA expression-based Stemness Score (RNAss), Epigenetically regulated RNA expression-based Stemness Score (EREG.EXPss), DNA methylation-based Stemness Score (DNAss), Epigenetically regulated DNA methylation-based Stemness Score (EREG-METHss), Differentially methylated probes-based Stemness Score (DMPss) and Enhancer Elements/DNA methylation-based Stemness Score (ENHss) based on the previous study (Malta et al., 2018).

### Gene Correlation Analysis

Pearson’s correlation analysis was employed to assess the correlation between mRNA expression of USP45 with 44 marker genes of RNA modification (m1A, m5C, m6A), 150 marker genes of five immune functions or processes (chemokine, receptor, MHC, immunoinhibitor, immunostimulator) and 60 types of immune checkpoint pathway-related genes (inhibitory and stimulatory) based on previous studies with all types of TCGA cancer samples by using Sangerbox (http://vip.sangerbox.com/) or TIMER2.0 (http://timer.cistrome.org/).

### Immune Infiltration Analysis

The stromal score, immune score, and ESTIMATE score of all tumor samples from the TCGA database were calculated by the ESTIMATE package. The XCell method of the IOBR package was used to calculate the infiltration of various types of immune cells based on gene expression signatures in TCGA tumor samples. The correlation between mRNA expression of USP45 and the above-stated scores, infiltration of immune cells were calculated by Pearson’s correlation test.

### Construction of USP45 Related Competitive Endogenous RNA Network and Protein-Protein Interactions Network

ENCORI database (https://starbase.sysu.edu.cn/index.php) was employed to predict the candidate lncRNAs and microRNAs of USP45 among different types of cancers. The selected lncRNAs should have a positive correlation with USP45 and a negative relationship with candidate microRNAs based on expression profiles to build up Construction of USP45 Related Competitive Endogenous RNA (ceRNA) network. The correlation among USP45, target lncRNAs, and microRNAs were calculated and depicted by Pearson’s correlation test using samples from TCGA samples *via* ENCORI database. STRING database (https://string-db.org/) was used to predict protein-protein interactions of UPS45 with other candidate proteins, and the Network and Protein-Protein Interactions (PPI) network was modified by Cytoscape 3.7.2 (https://cytoscape.org/).

### USP45 Associated Differentially Expressed Genes Analysis

The different types of TCGA tumor samples were divided into high and low expression groups by the cut-off value of the median of USP45 mRNA expression. The Limma package was employed to identify differentially expressed genes between the high and low UPS45 expression groups, based on the selecting criteria of adjusted *p* < 0.05 and |logFC| ≥ 1, which were considered to have a significant difference and defined as the thresholds for the screening of differential expression of genes. The GO function and pathway enrichment analysis of Differentially Expressed Genes (DEGs) were assessed by the ClusterProfiler package.

### Immunohistochemistry of Tissue Microarrays

The protein expression level of USP45 was detected by immunohistochemistry in COADREAD (*n* = 64), ESCA (*n* = 36), GBMLGG (*n* = 38), LIHC (*n* = 47), LUAD (*n* = 63), LUSC (*n* = 38), OSCC (*n* = 63), PAAD (*n* = 47), PRAD (*n* = 43), RCC (*n* = 78), TNBC (*n* = 42), non-TNBC (*n* = 38) and their paired paracancerous tissues. All of the tissue microarrays were purchased from were purchased from Outdo Biotech company (Shanghai, China). The procedure for immunohistochemistry refers to our previous study (Li et al., 2020). The results processing of immunohistochemistry was performed by Pannoramic MIDI automatic digital slide scanner (3DHISTECH Ltd., Budapest, Hungary). The intensity of immunohistochemistry staining was then quantitatively scored. On the one hand, the proportion score was quantified as followed: 0 represents 0% of tumor cells exhibited positive staining, one represents 0%–1% positive cells, two represents 2%–10% positive cells, three represents 11%–30% positive cells, four represents 31%–70% positive cells, and five represents 71%–100% positive cells. On the other hand, the intensity score was quantified as followed: 0 represents negative; one represents weak; two represents moderate; and three represents strong. The total score is calculated as the mean of the proportion score and the intensity score.

### Drug Sensitivity Analysis

The relationship between drug sensitivity and the expression of USP45 and its top correlated genes was analyzed by “drug sensitivity analysis” module on GSCALite database (http://bioinfo.life.hust.edu.cn/web/GSCALite/) based on the data of 265 small molecules from Genomics of Drug Sensitivity in Cancer (GDSC). The gene expression corresponds with the drug according to the Spearman correlation. Positive association indicates that the gene with high expression is treatment resistant, and vice versa.

### Statistical Analysis

*p* < 0.05 was considered statistically significant. The analysis and visualization of the data were done by above stated online databases and tools, R 3.6.4, Excel 2019, and Cytoscape 3.7.2.

## Results

### mRNA Expression Levels of USP45 in Different Types of Tumor Tissues and Cell Lines

**FIGURE 1:**
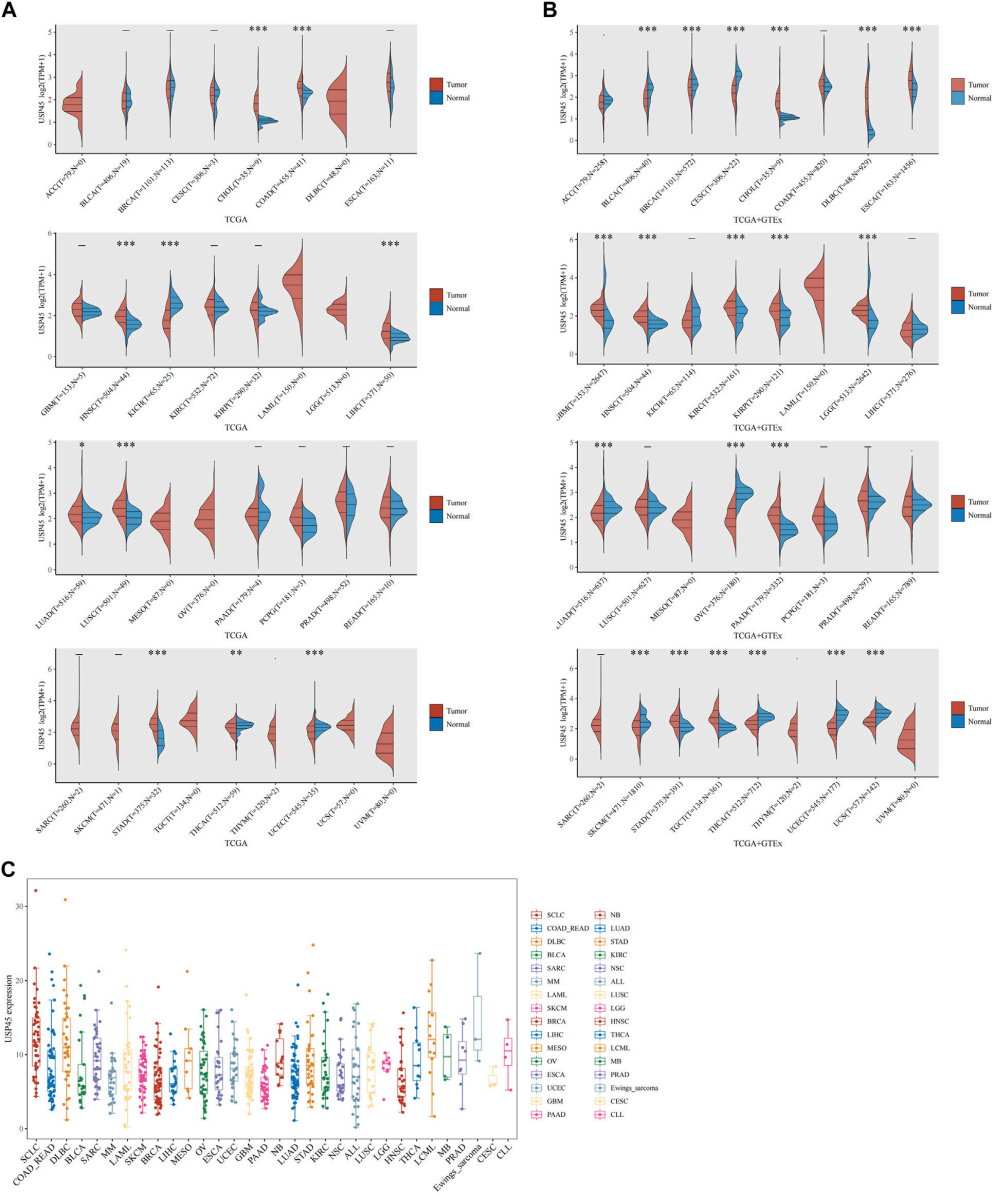
The expression analysis for USP45 in multiple types of cancer tissues and cell lines. RNA-seq data of 33 types of tumor and normal tissues were downloaded from The Cancer Genome Atlas (TCGA) database and GTEx database. **(A)** The expression of USP45 in 33 types of human cancer and normal tissues was analyzed based on TCGA database. **(B)** The expression of USP45 in 33 types of human cancer and normal tissues was analyzed based on TCGA and GTEx database. **(C)** The expression levels of USP45 in 946 types of tumor cell lines were analyzed based on the CCLE database. *p < 0.05, **p < 0.01, ***p < 0.001. The statistical difference of two groups was compared through the Wilcox test.

RNA-seq data of 33 types of tumor and normal tissues were downloaded from The Cancer Genome Atlas (TCGA) database and GTEx. The results of pan-cancer analysis showed that in the TCGA database, relative to the normal group, the mRNA expression levels of USP45 were significantly upregulated in cholangio carcinoma (CHOL), colon adenocarcinoma (COAD), head and neck squamous cell carcinoma (HNSC), liver hepatocellular cancer (LIHC), lung adenocarcinoma (LUAD), lung squamous cell carcinoma (LUSC), stomach adenocarcinoma (STAD), while downregulated in kidney chromophobe carcinoma (KICH), thyroid carcinoma (THCA) and uterine corpus endometrial carcinoma (UCEC). Since the TCGA database mainly contains tumor tissue data and relatively few data from normal tissues, the GTEx database mainly contains RNA-seq data from normal human tissues. Thus, in the TCGA and GTEx databases, the results of analysis showed that USP45 were significantly upregulated in CHOL, lymphoid neoplasm diffuse large B-cell lymphoma (DLBC), esophageal carcinoma (ESCA), glioblastoma multiforme (GBM), HNSC, KIRC, kidney renal papillary cell carcinoma (KIRP), brain lower grade glioma (LGG), pancreatic adenocarcinoma (PAAD), STAD, testicular germ cell tumors (TGCT), while downregulated in bladder urothelial carcinoma (BLCA), BRCA, cervical squamous cell carcinoma (CESC), LUAD, ovarian serious cystadenocarcinoma (OV), skin cutaneous melanoma (SKCM), THCA, UCEC, uterine carcinosarcoma (UCS) (Figures 1A,B). Next, we downloaded the gene expression data of the cell lines from the CCLE database (https://portals.broadinstitute.org/ccle/about), including 32 types of tumors, and constructed the expression profile of USP45 in 946 tumor cell lines (Figure 1C).

### Correlation Analysis of USP45 With Overall Survival and Recurrence-Free Survival in Patients With Different Types of Tumors

**FIGURE 2:**
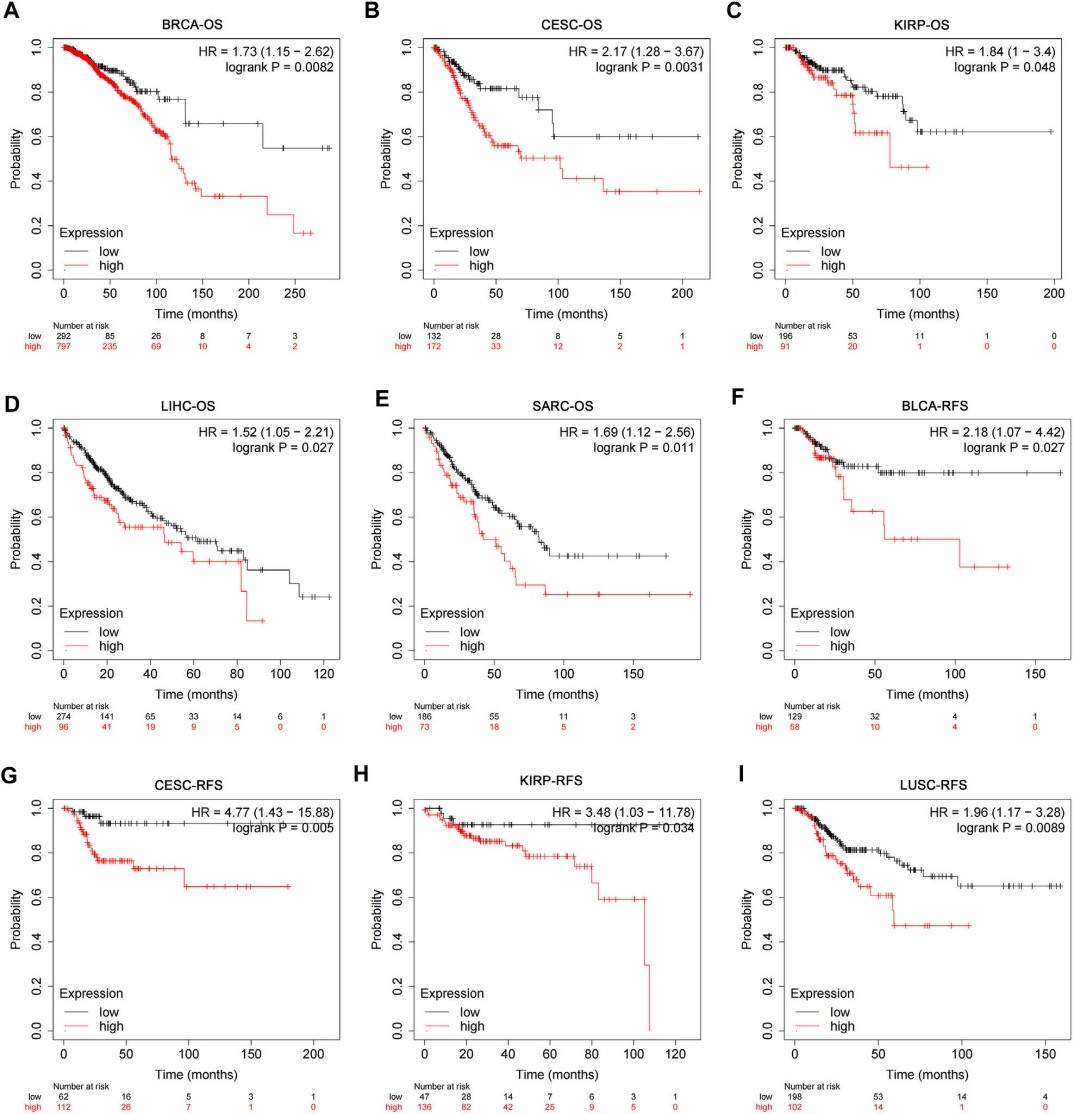
Correlation analysis of USP45 with OS and RFS in patients with different types of tumors. The RNA-seq data and corresponding clinical information of different types of tumors were downloaded from TCGA database. The expression levels of USP45 were negatively correlated with OS of patients with BRCA **(A)**, CESC **(B)**, KIRP **(C)**, LIHC **(D)**, and SARC **(E)**. The expression levels of USP45 were negatively correlated with RFS of patients with BLCA **(F)**, CESC **(G)**, KIRP **(H)**, and LUSC **(I)**.

The RNA-seq data and corresponding clinical information of different types of tumors were downloaded from TCGA database. The results of pan-cancer analysis showed that the expression of USP45 was negatively correlated with the overall survival (OS) of BRCA, CESC, KIRP, LIHC, and SARC (Figures 2A–E). In BLCA, CESC, KIRP, and LUSC, the expression of USP45 was negatively correlated with the recurrence-free survival (RFS) of patients (Figures 2F–I). In addition, although there is no statistical significance, there was a trend that the lower expression level of USP45, the longer OS and RFS of patients with BLCA, EAC, KIRC, PCPG, UCEC, LIHC, BRCA, HNSC, and LUAD (Supplementary Figure S1).

### Structural Variation, Gene Mutation and Gene Copy Number Variation of USP45 in Different Types of Tumors

**FIGURE 3:**
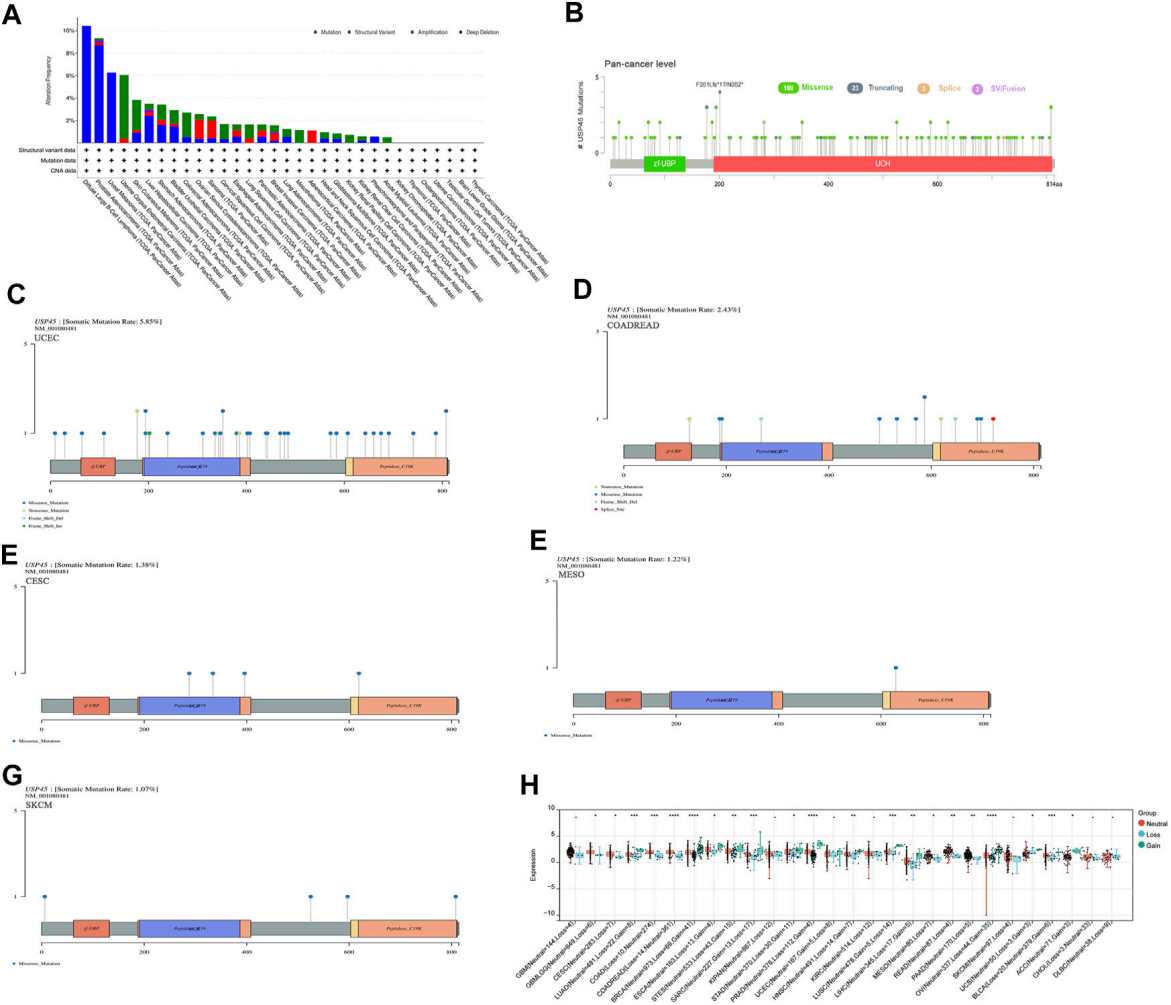
Structural variation, gene mutation and gene copy number variation of USP45 in different types of tumors. **(A)** Structural variation, gene mutation and gene copy number variation of USP45 in different types of tumors were analyzed based on the TCGA database. Somatic mutation rate and mutation type of USP45 at pan-cancer level **(B)** and UCEC **(C)**, COADREAD **(D)**, CESC **(E)**, MESO **(F)**, SKCM **(G)** were analyzed and shown in the figures. **(H)** The detailed information of gene copy number alteration of USP45 in different types of tumors were analyzed and shown in the figure. *p < 0.05, **p < 0.01, ***p < 0.001, ****p < 0.0001.

Structural variation, gene mutation and gene copy number alteration are important factors affecting gene expression and function, and are closely related to the activation of oncogenes or the inactivation of tumor suppressor genes (Malhotra et al., 2013; Schaefer et al., 2019; Visani et al., 2021). The results of pan-cancer analysis showed that the USP45 gene had a low frequency of structural variants, mainly present in PRAD and LIHC (Figure 3A). In different types of tumors, gene mutations of USP45 included missense, truncating, splice, and SV/Fusion, with F201Lfs*17/N202* mutations being the most common (Figure 3B). The frequency of mutations in USP45 was higher in UCEC, colorectal adenocarcinoma (COADREAD), CESC, MESO, and SKCM, at 5.85%, 2.43%, 1.38%, 1.22%, and 1.07%, respectively (Figures 3C–G). In other tumors, the frequency of mutations in USP45 is less than 1%. In addition, we further analyzed the gene copy number alteration of USP45 in detail and the results showed that deletion of USP45 gene fragments was more common in DLBA, PRAD, and UVM, while the frequency of amplification of USP45 gene fragment was higher in OV, SARC and ACC (Figures 3A,H).

### Correlation of USP45 With mRNA Methylation Modifications in Different Types of Tumors

**FIGURE 4:**
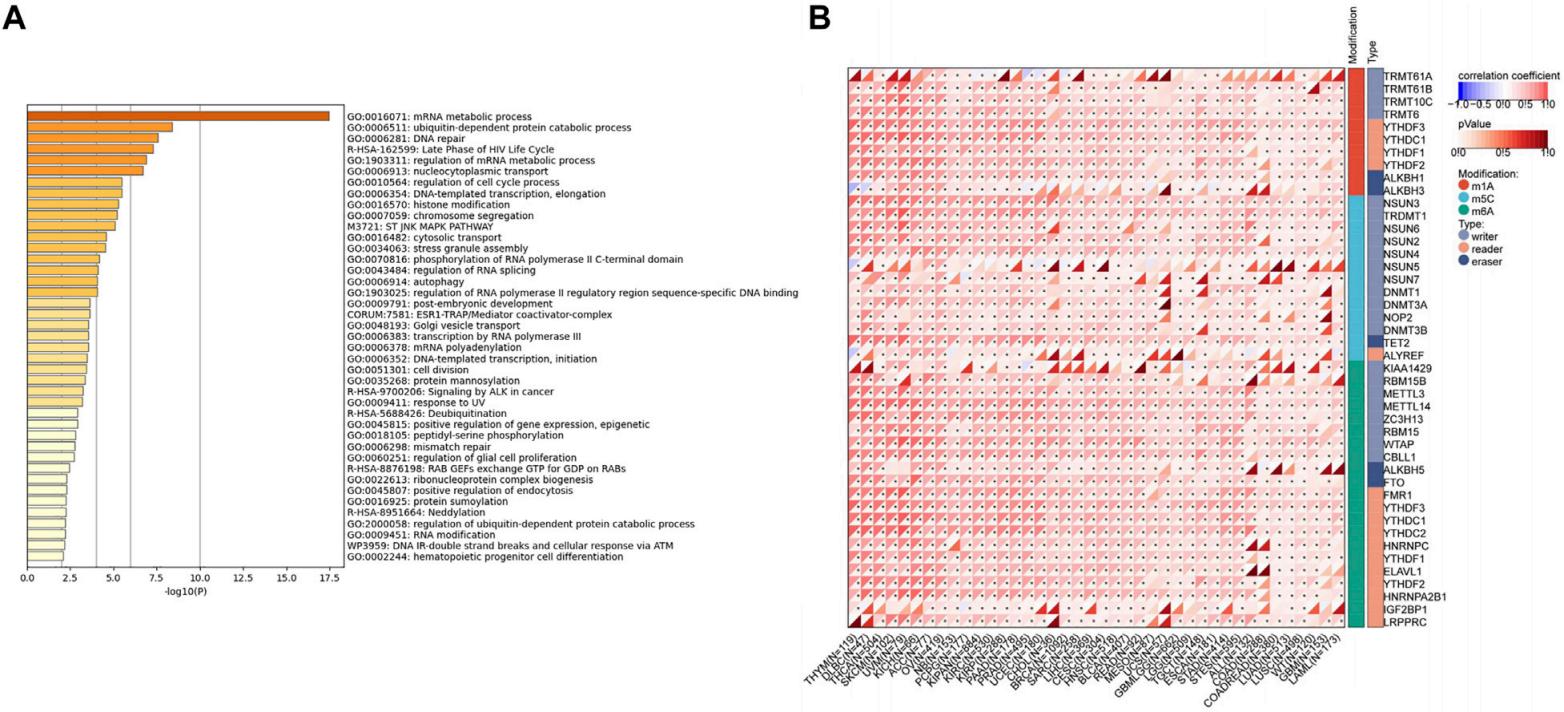
Correlation of USP45 with mRNA methylation modifications in different types of tumor. **(A)** The similar genes of USP45 were analyzed and obtained through the GEPIA website. Then, we performed GO analysis and KEGG pathway analysis on similar genes of USP45 *via* the metascape website. **(B)** The correlation of USP45 with Writer, Eraser, Reader of m6A RNA methylation, m5C RNA methylation, and m1A RNA methylation in different types of tumors was analyzed based on the TCGA database.

As the role of USP45 in tumorigenesis is unknown, similar genes of USP45 were analyzed through the GEPIA website to investigate the function of USP45. Then, we performed GO analysis and KEGG pathway analysis on similar genes of USP45 *via* the metascape website. The results showed that these genes were mainly involved in biological processes such as mRNA metabolism, protein ubiquitination, and DNA repair (Figure 4A). Since USP45 is a deubiquitinating enzyme, there is no doubt that USP45 can regulate protein ubiquitination (Conte et al., 2018). In addition, it has been reported that USP45 can regulate the ubiquitination of ERCC1 protein, thereby affecting the repair of UV-induced DNA damage (Perez-Oliva et al., 2015). However, the correlation between USP45 and mRNA metabolism is unclear. Modifications of mRNA methylation can regulate the process of mRNA translation and play an important role in mRNA metabolism (Yu et al., 2021). There are many types of RNA methylation modifications, with the most widely known being m6A RNA methylation, m5C RNA methylation, and m1A RNA methylation (An and Duan, 2022). In the process of RNA methylation, there are three important proteins, namely Writer, Eraser and Reader. Among them, the Writer protein is responsible for catalyzing the methylation process, while the Eraser catalyzes the demethylation process. After the formation of RNA methylation, Reader directly recognizes and binds the methylation site, so that the methylated RNA can play a specific role (Zhou et al., 2021). To further explore the relationship between USP45 and mRNA methylation modification in tumorigenesis and development, we analyzed the relationship between USP45 and Writer, Eraser, Reader of m6A RNA methylation, m5C RNA methylation, and m1A RNA methylation. For the m1A RNA methylation, we analyzed the correlation between USP45 and Writer (TRMT61A, TRMT61B, TRMT10C, and TRMT6), Reader (YTHDF3, YTHDC1, YTHDF1, and YTHDF2), Eraser (ALKBH1, ALKBH3). For the m5C RNA methylation, we analyzed the correlation between USP45 and Writer (NSUN3, TRDMT1, etc.), Reader (ALYREF), Eraser (TET2). For the m6A RNA methylation, we analyzed the correlation between USP45 and Writer (KIAA1429, RBM15B, etc.), Reader (FMR1, YTHDF3, etc.), Eraser (ALKBH5, FTO). The results showed that USP45 was positively related to the majority of Writer, Eraser, Reader of m6A RNA methylation, m5C RNA methylation, and m1A RNA methylation in pan-cancer (Figure 4B). Furthermore, we analyzed the prognostic value of DNA methylation expression levels of USP45 in pan-cancer, the results showed that cg08792129, cg10139443, cg13363689, cg21257892, cg16486502, cg26484090, cg25198784, cg25309811, cg27317327, cg10970399, cg13809693, and cg17635724 from USP45 had the highest DNA methylation levels and significant prognostic value (likelihood ratio (LR) test *p*-value < 0.05) in different types of tumors (Supplementary Table S1).

### Correlation of USP45 With Tumor Heterogeneity in Different Types of Tumors

**FIGURE 5:**
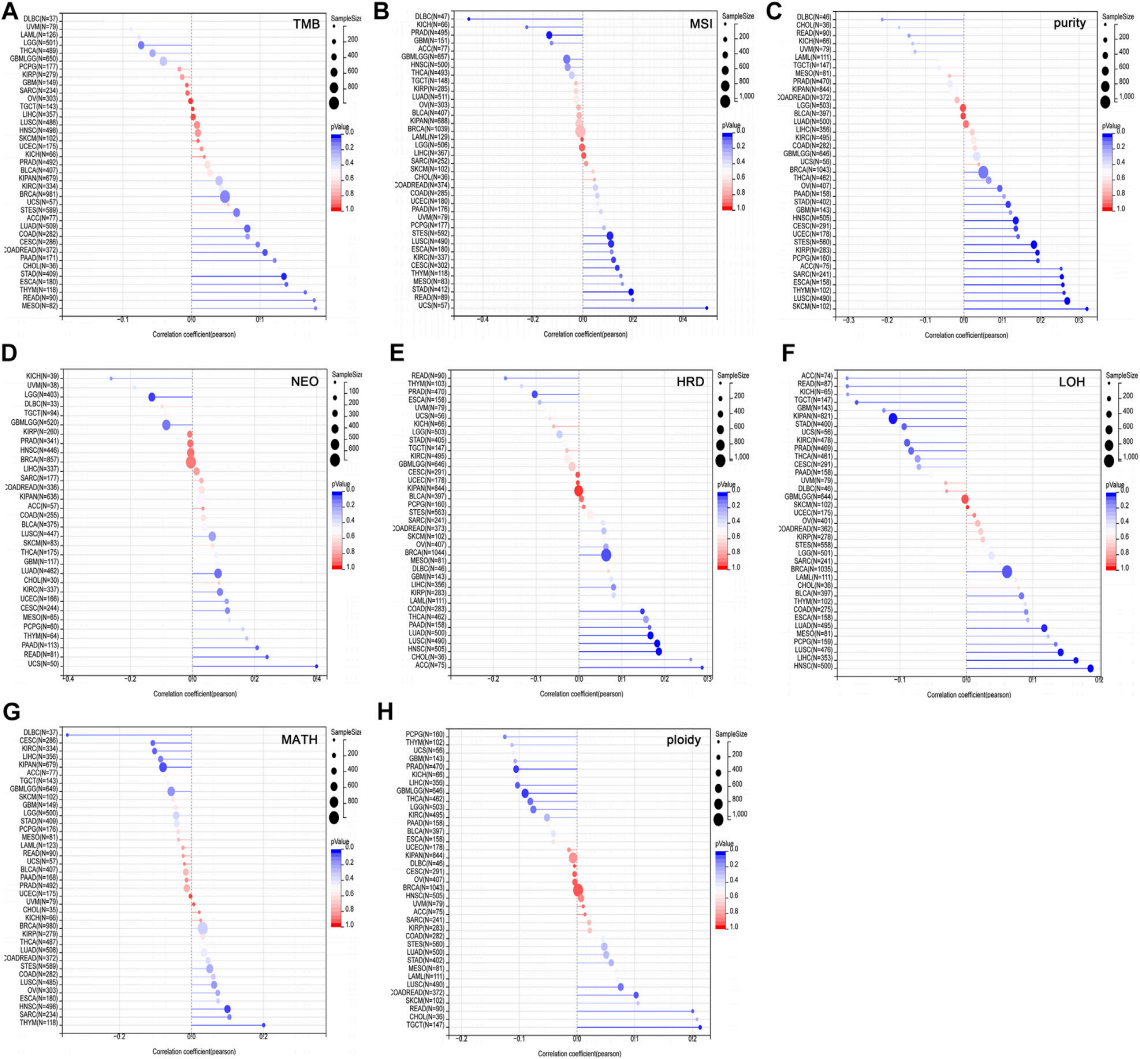
Correlation of USP45 with tumor heterogeneity in different types of tumors. Tumor heterogeneity includes tumor mutation burden (TMB), microsatellite instability (MSI), tumor purity, neoantigen (NEO), homologous recombination deficiency (HRD), loss of heterozygosity (LOH), mutant-allele tumor heterogeneity (MATH), tumor ploidy. The correlation between USP45 and TMB **(A)**, MSI **(B)**, tumor purity **(C)**, NEO **(D)**, HRD **(E)**, LOH **(F)**, MATH **(G)**, and ploidy **(H)** were analyzed based on TCGA database.

Tumor heterogeneity is one of the characteristics of malignant tumors, that is, after multiple divisions and proliferation, their progeny undergo molecular biology or genetic changes, leading to differences in tumor growth rate, invasion ability, drug sensitivity, and prognosis, etc. Therefore, the greater the tumor heterogeneity, the worse the treatment effect and prognosis of the patient (McGranahan and Swanton, 2017). Tumor heterogeneity includes tumor mutation burden (TMB), microsatellite instability (MSI), tumor purity, neoantigen (NEO), homologous recombination deficiency (HRD), loss of heterozygosity (LOH), mutant-allele tumor heterogeneity (MATH), tumor ploidy. MSI occurs due to functional defects in DNA mismatch repair in tumor tissue. The phenomenon of MSI with defective DNA mismatch repair is an important clinical tumor marker (Baretti and Le, 2018). TMB is highly correlated with the efficacy of PD-1/PD-L1 inhibitors in tumor immunotherapy (Strickler et al., 2021). In addition to tumor cells, tumor tissues also contain non-tumor cells such as immune cells, stromal cells, and interstitial cells, which together affect the occurrence and development of tumors. Tumor purity is significantly related to the clinical characteristics, genome expression and biological characteristics of tumor patients (Zhang et al., 2017). The influence of tumor purity should be fully considered when analyzing tumor samples during the research process. NEO, a tumor-specific antigen derived from nonsynonymous mutations, is a very attractive target for tumor immunotherapy and are abundantly expressed in tumor cells with strong immunogenicity and tumor heterogeneity (Peng et al., 2019). As a new tumor immunotherapy approach, vaccines developed against neoantigens have been used in clinical trials for various solid tumors (Blass and Ott, 2021). HRD can lead to specific, quantifiable, and stable genomic changes, which is a key indicator of tumor treatment and prognosis (Hoppe et al., 2018). Clinical studies have confirmed that HRD status is highly correlated with platinum-based chemotherapy drugs and PARP inhibitors (Chopra et al., 2020). LOH is ubiquitous in tumors and closely associated with the inactivation of tumor suppressor genes (Thiagalingam et al., 2002). MATH is used to evaluate the overall statistical distribution of genetic heterogeneity in tumor samples, and the larger the MATH value, the higher the tumor heterogeneity (Hou et al., 2020). Ploidy is a hallmark of cancer and closely related to chromosomal instability involved in cancer development (Zhao et al., 2021). To evaluate the correlation between USP45 and tumor heterogeneity, we analyzed the RNA-seq data in the TCGA database, and the results showed that USP45 was positively related to TMB in COADREAD and STAD (Figure 5A), positively related to MSI in CESC, STES, STAD, KIRC, LUSC, and UCS, while negatively related to MSI in PRAD and DLBC (Figure 5B), positively related to tumor purity in CESC, ESCA, STES, SARC, KIRP, STAD, HNSC, LUSC, THYM, PCPG, SKCM, and ACC (Figure 5C), positively related to NEO in READ and UCS, while negatively related to NEO in LGG (Figure 5D), positively related to HRD in LUAD, COAD, BRCA, HNSC, LUSC, and ACC, while negatively related to HRD in PRAD (Figure 5E), positively related to LOH in LUAD, HNSC, LUSC, and LIHC, while negatively related to LOH in KIPAN and TGCT (Figure 5F), positively related to MATH in HNSC and THYM, while negatively related to MATH in KIPAN and DLBC (Figure 5G), positively related to ploidy in COADREAD and TGCT, while negatively related to ploidy in GBMLGG and PRAD (Figure 5H).

### Correlation of USP45 With Tumor Stemness in Different Types of Tumors

**FIGURE 6:**
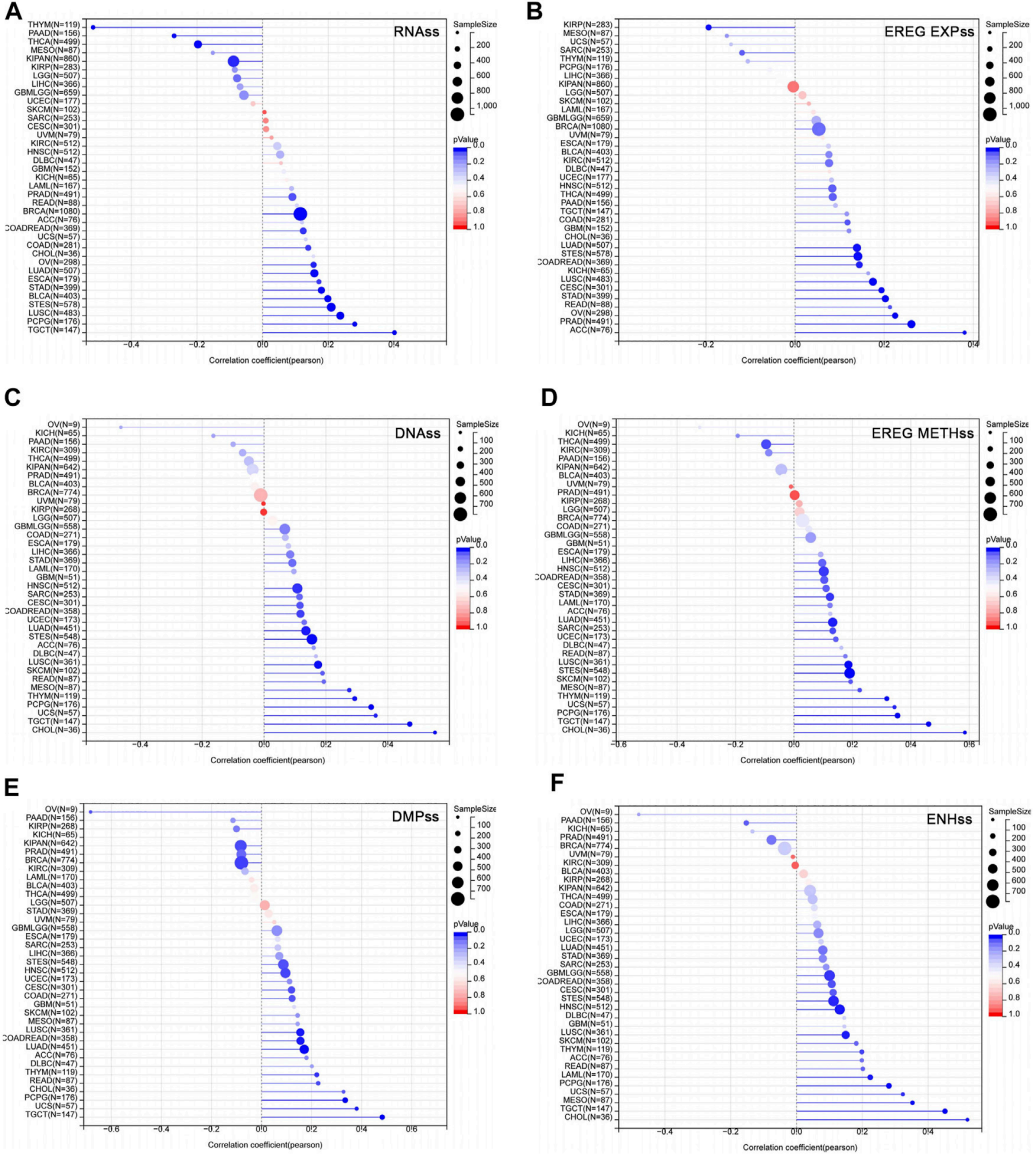
Correlation of USP45 with tumor stemness in different types of tumors. The stemness index included RNAss, EREG EXPss, DNAss, EREG METHss, DMPss, and ENHss based on mRNA expression and DNA methylation. The correlation between USP45 and tumor stemness index, including RNAss **(A)**, EREG EXPss **(B)**, DNAss **(C)**, EREG METHss **(D)**, DMPss **(E)** and ENHss **(F)** were analyzed based on the TCGA database.

Studies have shown that the acquisition of stem cell-like characteristics is the main drivers of tumor progression (Primeaux et al., 2022). The stemness index refers to the similarity between tumor cells and stem cells, including RNAss, EREG EXPss, DNAss, EREG METHss, DMPss, and ENHss based on mRNA expression and DNA methylation. These stemness indices range from 0 to 1, where 0 means that the tumor cells are less similar to stem cells, and 1 means that the tumor cells are more similar to stem cells. We analyzed the correlation between USP45 and tumor stemness indices in different types of tumors. The results showed that in the majority types of tumors, the expression levels of USP45 were positively correlated with the stemness indices, indicating that USP45 was closely related to the formation of tumor stemness (Figure 6).

### Correlation of USP45 With Tumor-Infiltrating Immune Cells, Immune Checkpoint Molecules and Immune Regulators in Different Types of Tumors

**FIGURE 7:**
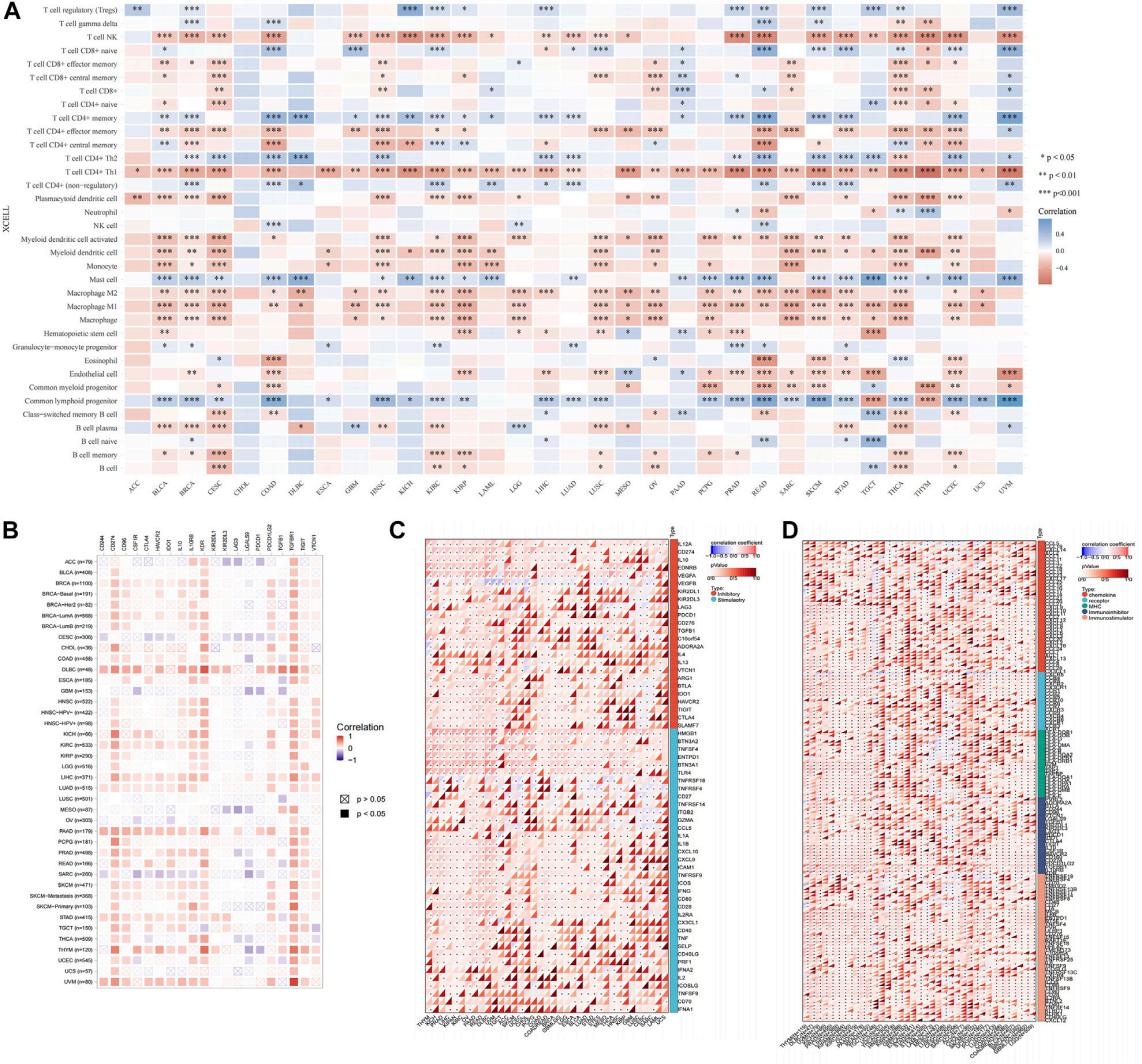
Correlation of USP45 with tumor immunity in different types of tumors. Based on the TCGA database, the xCELL algorithm was used to analyze the correlation of USP45 with tumor-infiltrating immune cells **(A)**, immune checkpoint molecules **(B,C)** and immune regulators **(D)**. *p < 0.05, **p < 0.01, ***p < 0.001.

The type and number of immune cells in the tumor microenvironment are closely related to the development of tumors and the effect of tumor immunotherapy (Lei et al., 2020). We analyzed the correlations between USP45 and 35 types of tumor-infiltrating immune cells in tumors. The results showed that the expression level of USP45 was negatively correlated with the infiltration levels of NK cells, Th1 cells, macrophages, and dendritic cells in the majority types of tumors (Figure 7A). In addition, the expression level of USP45 was negatively correlated with StromalScore, ImmuneScore, and ESTIMATEScore in CESC, LAML, STES, SARC, KIRP, UCEC, LUSC, THCA, OV, TGCT, PCPG, SKCM, and ACC (Table 1). Furthermore, we also assessed the correlation between USP45 and 60 types of immune checkpoint molecules and 150 types of immune regulators. The results showed that in the majority types of tumors, the expression of USP45 was positively correlated with many immune checkpoint molecules and immune regulators such as PD-L1 (Figures 7B–D). The above results suggested that USP45 was closely related to tumor immunity and might affect the effectiveness of tumor immunotherapy.

**TABLE 1:** The relationship between the USP45 gene expression and immune score.

**Cancers Immune score**	**StromalScore**		**ImmuneScore**		**Estimate Score**	
	**Correlation**	**P value**	**Correlation**	***p* value**	**Correlation**	***p* value**
GBM (N = 152)	−0.15	0.07	−0.14	0.07	−0.16	0.05
LGG (N = 504)	0.03	0.57	0.01	0.88	0.01	0.76
CESC (N = 291)	−0.24	0.00	−0.39	0.00	−0.36	0.00
LUAD (N = 500)	0.02	0.61	−0.03	0.46	−0.01	0.87
COAD (N = 282)	0.08	0.16	0.08	0.17	0.09	0.13
COADREAD (N = 373)	0.10	0.06	0.10	0.05	0.11	0.03
LAML (N = 149)	−0.29	0.00	−0.34	0.00	−0.35	0.00
BRCA (N = 1077)	−0.02	0.42	−0.10	0.00	−0.08	0.01
ESCA (N = 181)	−0.10	0.16	−0.15	0.04	−0.14	0.06
STES (N = 569)	−0.19	0.00	−0.23	0.00	−0.23	0.00
SARC (N = 258)	−0.24	0.00	−0.33	0.00	−0.32	0.00
KIRP (N = 285)	−0.19	0.00	−0.25	0.00	−0.25	0.00
KIPAN (N = 878)	0.07	0.05	−0.04	0.25	0.01	0.76
STAD (N = 388)	−0.10	0.06	−0.11	0.03	−0.11	0.03
PRAD (N = 495)	−0.07	0.15	−0.12	0.01	−0.10	0.02
UCEC (N = 178)	−0.22	0.00	−0.36	0.00	−0.33	0.00
HNSC (N = 517)	−0.02	0.62	−0.22	0.00	−0.14	0.00
KIRC (N = 528)	0.04	0.40	−0.09	0.03	−0.05	0.29
LUSC (N = 491)	−0.22	0.00	−0.26	0.00	−0.26	0.00
THYM (N = 118)	0.14	0.14	−0.32	0.00	−0.16	0.08
LIHC (N = 363)	−0.03	0.53	−0.13	0.02	−0.09	0.10
THCA (N = 503)	−0.17	0.00	−0.29	0.00	−0.26	0.00
MESO (N = 85)	0.09	0.41	−0.19	0.08	−0.09	0.42
READ (N = 91)	0.12	0.25	0.18	0.09	0.15	0.16
SKCM-M (N = 351)	−0.06	0.25	−0.11	0.03	−0.10	0.06
SKCM (N = 452)	−0.07	0.14	−0.12	0.01	−0.11	0.02
PAAD (N = 177)	0.20	0.01	0.14	0.07	0.18	0.01
OV (N = 417)	−0.12	0.01	−0.15	0.00	−0.15	0.00
TGCT (N = 132)	−0.40	0.00	−0.08	0.33	−0.24	0.01
PCPG (N = 177)	−0.23	0.00	−0.16	0.03	−0.20	0.01
SKCM-P (N = 101)	−0.28	0.00	−0.38	0.00	−0.36	0.00
UVM (N = 79)	−0.11	0.35	−0.05	0.67	−0.07	0.55
UCS (N = 56)	−0.05	0.74	−0.17	0.22	−0.16	0.23
BLCA (N = 405)	−0.12	0.02	−0.05	0.34	−0.09	0.06
ACC (N = 77)	−0.30	0.01	−0.34	0.00	−0.33	0.00
KICH (N = 65)	0.07	0.59	−0.03	0.79	0.01	0.91
CHOL (N=36)	0.16	0.36	0.08	0.62	0.10	0.56
DLBC (N = 46)	0.18	0.23	0.30	0.04	0.32	0.03

### Construction of the ceRNA Regulatory Network for USP45 in Different Types of Tumors

**FIGURE 8:**
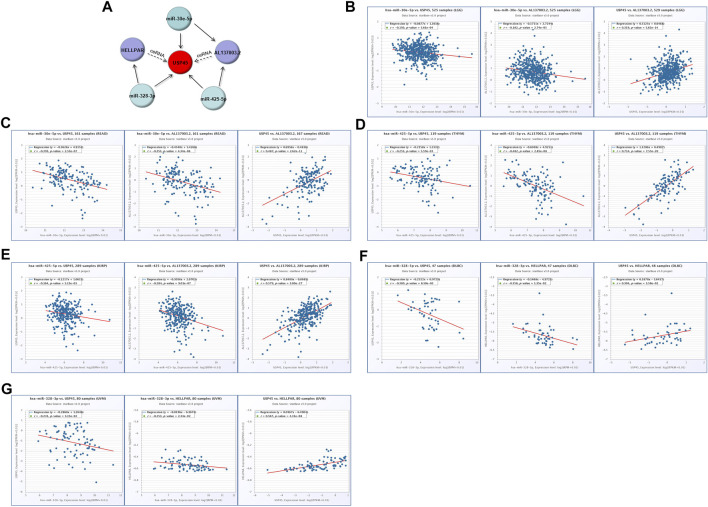
Construction of the ceRNA network for USP45. **(A)** The ceRNA network for USP45 was analyzed using the ENCORI database, and the analysis results were visualized with cytoscape software. In LGG **(B)** and READ **(C)**, the correlations among USP45, miR-30e-5p with AL137003.2 were analyzed. In THYM **(D)** and KIRP **(E)**, the correlation among USP45, miR-425-5p with AL137003.2 was analyzed. In DLBC **(F)** and UVM **(G)**, the correlations among USP45, miR-328-3p with HELLPAR were analyzed.

Our results have shown that USP45 is upregulated in the majority types of tumors. However, no study has yet investigated the upstream regulation mechanism of USP45. It is well known that the ceRNA mechanism is one of the important molecular mechanisms for regulating gene expression (Qi et al., 2015). Briefly, if both lncRNA and mRNA are targets of a miRNA, then lncRNA may compete for binding to miRNA, thereby upregulating mRNA expression (Braga et al., 2020). Through the ENCORI database, we found that both USP45 and lncRNA AL137003.2 may be the targets of miR-30e-5p and miR-425-5p, while USP45 and lncRNA HELLPAR may be the targets of miR-328-3p (Tables 2, 3). Therefore, we constructed the ceRNA network for USP45 (including USP45-miR-328-3p-HELLPAR, USP45-miR-425-5p-AL137003.2, USP45-miR-30e-5p-AL137003.2) (Figure 8A). Then, in order to verify the possibility of the above ceRNA mechanism, we further analyzed the correlation between lncRNA, miRNA, and mRNA. The results showed that in LGG, READ, COAD, and PRAD, the expressions of USP45 and AL137003.2 were negatively correlated with miR-30e-5p, while USP45 was positively correlated with AL137003.2 (Figures 8B,C; Supplementary Figures S2A,B). In KICH, THYM, SARC, LGG, KIRP, THCA, PRAD, the expressions of USP45 and AL137003.2 were negatively correlated with miR-425-5p, while USP45 was positively correlated with AL137003.2 (Figures 8D,E; Supplementary Figures S2G–H). In DLBC、UVM、LGG, the expressions of USP45 and HELLPAR were negatively correlated with miR-328-3p, while USP45 was positively correlated with HELLPAR (Figures 8F,G; Supplementary Figure S2H). These results suggested that the above ceRNA regulatory mechanism for USP45 might be feasible.

**TABLE 2:** The predicted miRNAs which may target to the 3’UTR of USP45 mRNA.

**mRNA**	**miRNA**	**Binding sites**	**Alignment**
USP45	miR-30e-5p	chr6:99881228-99881256[−]	Target: 5' uaaauaaa**UAA**UA**AUGUUUAC**u 3' miRNA : 3' gaagguca**GUU**CC**UACAAAUG**u 5'
USP45	miR-425-5p	chr6:99882337-99882359[−]	Target: 5' au**AAUGG**CU**A**C**U**----**UC**A**GUGUCAU**a 3' miRNA : 3' ag**UUGCC**--C**U**C**A**CU**AG**--**CACAGUA**a 5'
USP45	miR-328-3p	chr6:99883029-99883035[−]	Target: 5' cuuaaaaucaucuc**AGGGCCA**u 3' miRNA : 3' ugccuucccgucuc**UCCCGGU**c 5'

**TABLE 3:** The predicted miRNAs which may target to the lncRNAs.

**lncRNA**	**miRNA**	**Binding sites**	**Alignment**
AL137003.2	miR-30e-5p	chr6:16765482-16765503[+]	Target: 5' ugcgccuuuugcu**GUGUUUAC**a 3' miRNA: 3' gaaggucaguucc**UACAAAUG**u 5'
AL137003.2	miR-425-5p	chr6:16765441-16765463[+]	Target: 5' acuuaauuucauu**UGGUGUCAU**a 3' miRNA: 3' aguugcccucacu**AGCACAGUA**a 5'
HELLPAR	miR-328-3p	chr12:102782543-102782563[+]	Target: 5' uuugauccc**CA**--**AAAGGGCCA**g 3' miRNA: 3' ugccuuccc**GU**C**UCUCCCGGU**c 5'

### Construction of the Protein-Protein Interaction Network and Downstream Regulatory Network for USP45

**FIGURE 9:**
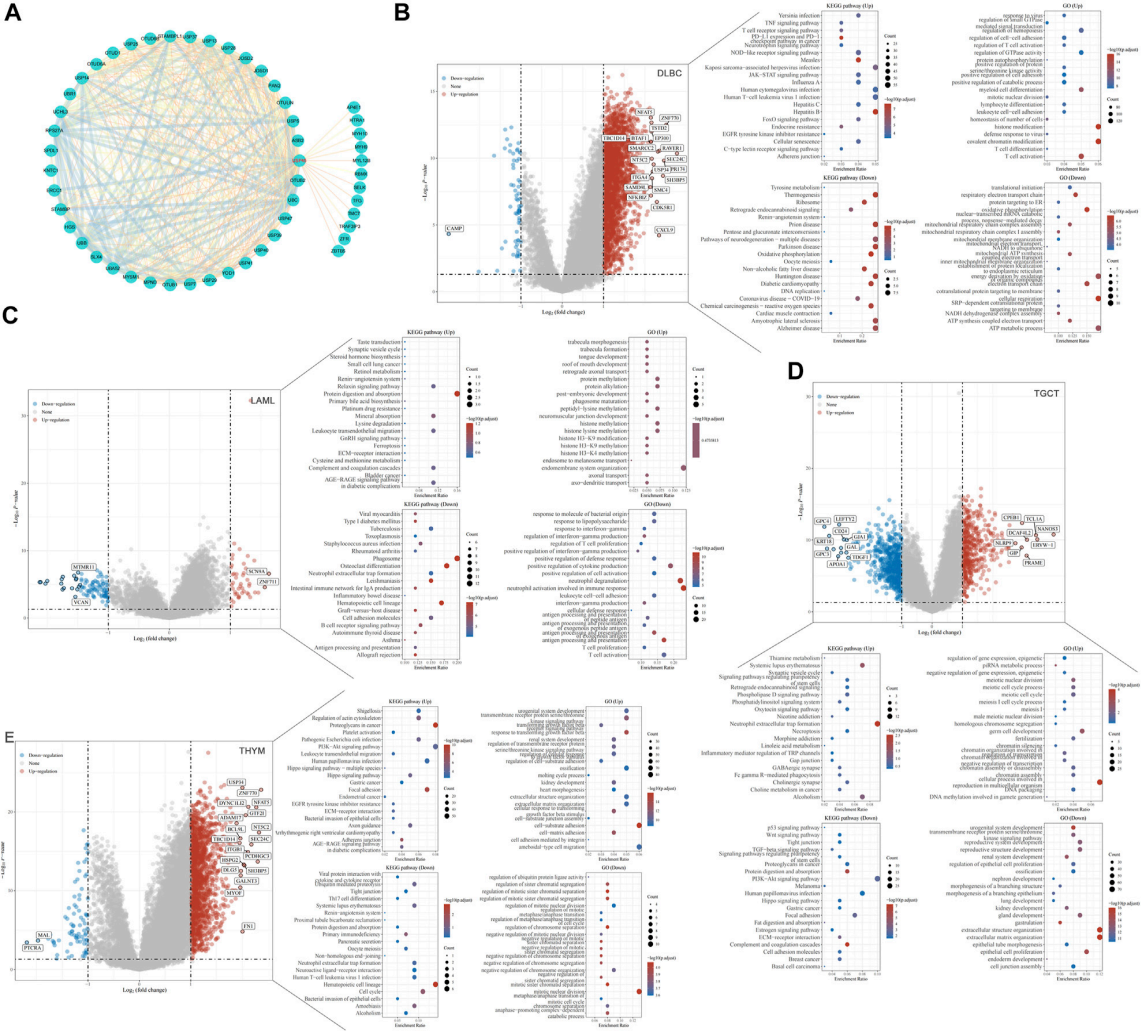
Construction of the protein-protein interaction network and downstream regulatory network for USP45. **(A)** Proteins that may interact with USP45 were analyzed based on the string database, and then the interaction network was visualized using cytoscape software. In addition, the thickness of the line between proteins represents the score of prediction. Based on the RNA-seq data in TCGA and GTEx databases, DLBC **(B)**, LAML **(C)**, TGCT **(D)** and THYM **(E)** were divided into USP45 high expression and low expression groups according to the mean of mRNA expression levels. Then, the upregulated and downregulated genes were screened out using the Limma package of R software, and subjected to GO analysis and KEGG analysis using the ClusterProfiler package of R software, respectively.

Proteins are the executors of vital activities, and their functions mainly depend on the interactions between the proteins. Based on the STRING database, we analyzed proteins that may interact with USP45, and then constructed the protein-protein interaction network for USP45 by cytoscape software (Figure 9A). Among them, the interaction of USP45 with ERCC1, SPDL1, MYH9, MYH10, MYL12B, ZFR, RBMX has been confirmed (Sowa et al., 2009; Perez-Oliva et al., 2015; Conte et al., 2018). The molecular mechanism of USP45 in the occurrence and development of tumors was still unclear. To explore the mechanism of USP45 in different types of tumors, we constructed the downstream regulatory network for USP45. Based on the RNA-seq data in TCGA and GTEx databases, the tumors were divided into USP45 high expression and low expression groups according to the mean of mRNA expression levels. Then, the up- and down-regulated genes were screened out using the Limma package of R software, and subjected to GO analysis and KEGG analysis using the ClusterProfiler package of R software, respectively (Figures 9B–E, Supplementary Figures S3–S7). These results suggested that USP45 might exert diverse mechanisms by up- or down-regulating certain genes in different types of tumors. For example, KEGG pathway analysis showed that USP45 might regulate PD-L1 expression and PD-1 pathway, and GO analysis showed that USP45 might regulate T cell activation and differentiation through these upregulated genes in DLBC. The results further validated and explained the conclusion that USP45 was closely associated with tumor immunity. In conclusion, the protein-protein interaction network and downstream regulatory network for USP45 could provide a clear and comprehensive insight into the molecular mechanisms of USP45 in different types of tumors.

### Detection of USP45 Protein Expression by Immunohistochemistry of Tumor Tissue Microarray

**FIGURE 10:**
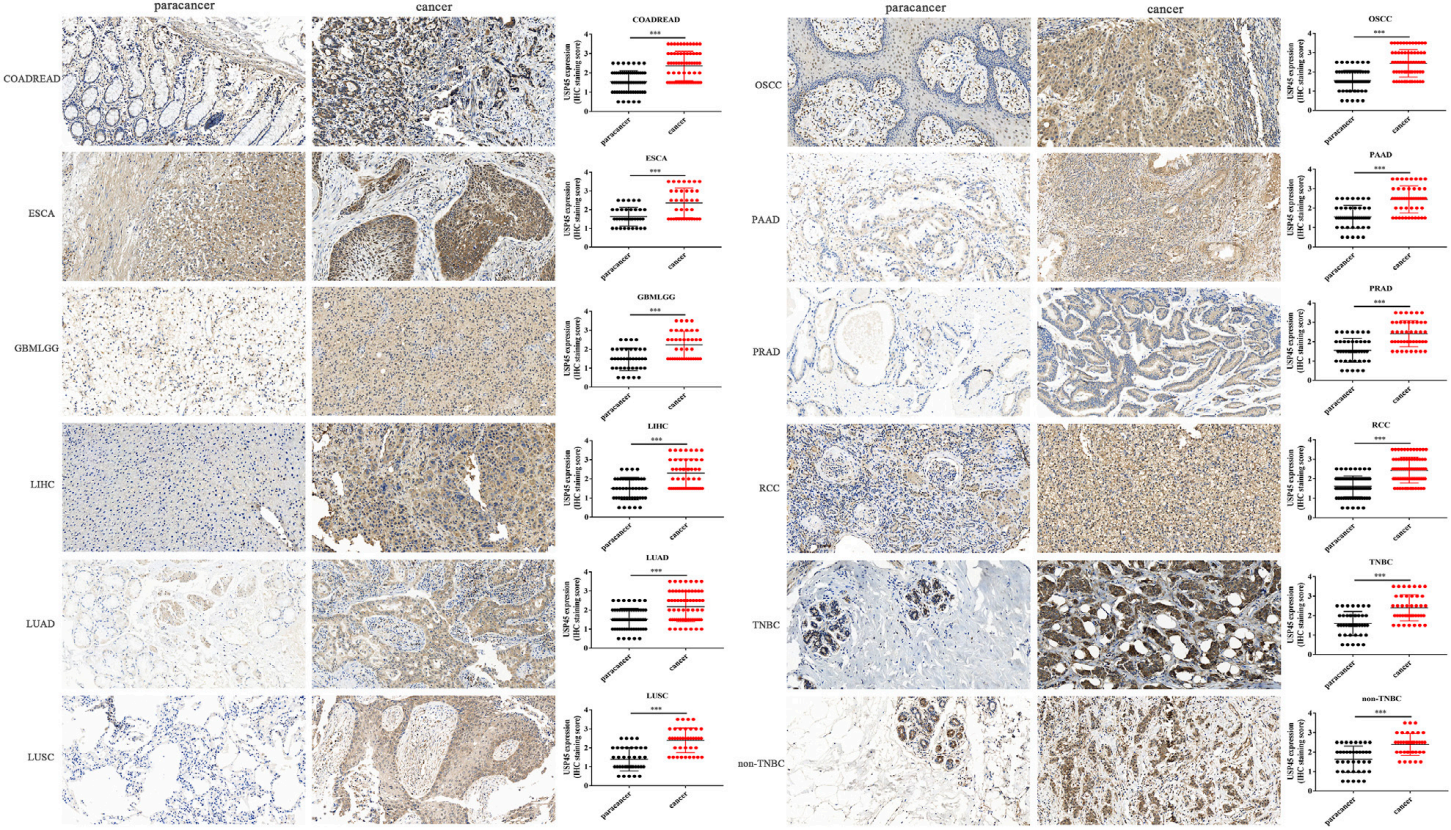
The protein expression levels of USP45 in 12 types of tumors and paired paracancerous tissues. The protein expression level of USP45 was detected and quantified by immunohistochemistry in COADREAD, ESCA, GBMLGG, LIHC, LUAD, LUSC, OSCC, PAAD, PRAD, RCC, TNBC, non-TNBC and their paired paracancerous tissues. ***p < 0.001.

Through pan-cancer analysis, we found that the mRNA expression levels of USP45 were upregulated in the majority types of tumors, suggesting that USP45 may be involved in tumorigenesis and development as an putative oncogene. To further validate our conclusions, we analyzed the protein expression levels of USP45 in 12 types of tumor and their paired paracancerous tissues, including COADREAD, ESCA, GBMLGG, LIHC, LUAD, LUSC, oral squamous cell carcinoma (OSCC), PAAD, PRAD, renal cell carcinoma (RCC), triple-negative breast cancer (TNBC), non-triple-negative breast cancer (non-TNBC) using immunohistochemistry. The results showed that relative to paracancerous tissues, USP45 was significantly upregulated in COADREAD, ESCA, GBMLGG, LIHC, LUAD, LUSC, OSCC, PAAD, PRAD, RCC, TNBC, non-TNBC (Figure 10). This result was consistent with the pan-cancer analysis. Therefore, we proposed that USP45 may be involved in tumor development as a putative oncogene.

### Analysis of Drug Sensitivity

USP45 and its top correlated genes were selected for drug sensitivity analysis, and the results (Supplementary Figure S8) showed that these genes with high expression are significantly associated with the resistance to a variety of anti-tumor drugs, such as Trametinib, selumetinib, Dabrafenib, Lapatinib, Erlotinib, Gefitinib, Docetaxel, and 17-AAG, indicating the oncogenic role of them. By contrast, their expressions were significantly associated with sensitivity of enriched anti-tumor drugs, such as PDPK1 inhibitor BX-912, Small Molecule NPK76−II−72−1, mitotic motor protein inhibitor Ispinesib, and chemotherapeutic drugs Methotrexate and 5−Fluorouracil, highlighting the potential clinical therapeutic strategies for USP45 overexpressed cancer patients.

## Discussion

The ubiquitin-proteasome pathway is an important regulatory system for protein degradation in the cell (Sharma et al., 2021). Through polyubiquitination and degradation of substrate proteins, a wide range of cellular activities can be regulated, including gene transcription, cell cycle, immune response, tumor growth and inflammatory processes (Brehm and Krüger, 2015; Collins et al., 2017). This pathway is a dynamic bidirectional protein modification regulatory system in which the substrate is ubiquitinated by the ubiquitin ligase system (E1-E2-E3) and the DUB family is responsible for the reverse regulation of protein degradation by hydrolysing the ester, peptide or isopeptide bonds at the carboxyl terminus of ubiquitin, hydrolysing the ubiquitin molecule specifically from the ubiquitin-linked protein or precursor protein, thereby affecting protein function.

The USPs family has the largest number of members (more than 50) and the most diverse structures among the known deubiquitinating enzymes (Li and Reverter, 2021). Many studies have demonstrated that USPs can participate in the occurrence and development of tumors by regulating multiple signaling pathways. For example, USP2a can catalyze the deubiquitination of MDM2, which in turn enhances p53 degradation (Stevenson et al., 2007). USP4 activates the Wnt/β-catenin signaling pathway by promoting the deubiquitination of β-catenin (Yun et al., 2015). USP37 interacts directly with MYC to promote its deubiquitination and maintain its protein stability (Pan et al., 2015). In recent years, studies have found that USPs are closely associated with tumor immune escape. In LIHC and NSCLC, USP22 acts as a deubiquitinating enzyme for PD-L1, inhibiting the ubiquitin-proteasome degradation pathway of PD-L1 and thus leading to tumor immune escape (Huang et al., 2019; Wang et al., 2020). In OSCC, USP9X can bind to PD-L1 to induce its deubiquitination, ultimately causing tumor cells to escape T cell attack (Jingjing et al., 2018). As a member of the USPs family, the mechanism of USP45 in tumorigenesis and development remains unclear.

In this study, we comprehensively explored the role of USP45 in different types of tumor through pan-cancer analysis for the first time. Through bioinformatics analysis combined with immunohistochemistry of tumor tissue microarray, we confirmed that USP45 acted as a putative oncogene in the majority types of tumors and was negatively correlated with the prognosis of tumor patients. And our results showed that the role of USP45 in the development of tumor maybe complex and variety. Studies have confirmed that tumor stemness is positively correlated with tumor cell proliferation and metastasis (Su et al., 2019; Prasad et al., 2020). In the present study, we demonstrated that the expression level of USP45 in the majority types of tumors was positively correlated with the tumor stemness index, suggesting that upregulation of USP45 may promote the proliferation and metastasis of tumor cells. In addition, we found that the expression levels of USP45 are positively correlated with tumor heterogeneity, which is an important cause of drug resistance and a challenge for precision tumor medicine. Therefore, our findings suggest that USP45 can be used as a biomarker of drug effectiveness in tumor treatment. Tumor immunotherapy mainly works by rebuilding the patient’s immune system, and then relies on the immune system to attack tumor cells. Therefore, the effect of tumor immunotherapy requires the presence of sufficient and effective immune cells in the tumor microenvironment (Zhang and Zhang, 2020). Through analysis, we found that USP45 is closely related to the infiltration level of tumor killer cells such as NK cells, macrophages, and dendritic cells in the tumor microenvironment, as well as immune checkpoints such as PD-L1, suggesting that USP45 can be used as a target of tumor immunotherapy to enhance the effect of tumor immunotherapy.

More importantly, we constructed the upstream and downstream regulatory networks of USP45, which provided a reference for revealing the mechanism of USP45 in tumorigenesis and development. The ceRNA mechanism plays an important role in regulating gene expression, the mechanism of which is more complex than miRNA (Smillie et al., 2018). In the ceRNA network, since lncRNAs and mRNAs are the target genes of miRNAs, their expression levels are negatively correlated with miRNAs, while there is a positive correlation between lncRNAs and mRNAs. Through the ENCORI database, we got many predicted ceRNA networks for USP45. However, based on the correlation of lncRNA-mRNA-miRNA, we finally constructed ceRNA networks for USP45, including USP45-miR-328-3p-HELLPAR, USP45-miR-425-5p-AL137003.2, USP45-miR-30e-5p-AL137003.2. As a deubiquitinating enzyme, the mechanism of USP45 in tumors must be achieved through its catalytic substrate. Therefore, the construction of protein-protein interaction network for USP45 would help to deeply understand its mechanism. Furthermore, we constructed the downstream regulatory network for USP45 in different types of tumors. For example, USP45 is associated with PD-L1 expression, PD-1 checkpoint pathway, TNF signaling pathway, and JAK/STAT signaling pathway in DLBC. In TGCT, USP45 is associated with P53 signaling pathway, Wnt signaling pathway, TGF-β signaling pathway, and PI3K/Akt signaling pathway. In DLBC and LAML, USP45 is associated with differentiation, proliferation, and activation of T cells. These results suggest that USP45 may have common and specific mechanisms in different types of tumors, which will help to further understand the mechanism of USP45 in tumorigenesis and development.

In this study, we firstly explored the putative oncogenic role of USP45 in pan-cancer, including the correlation between USP45 and tumor prognosis, mRNA methylation, tumor heterogeneity, tumor stemness, tumor immunity. And the construction of ceRNA regulatory network, protein-protein interaction network and downstream regulatory network for USP45 would also help to understand and provide insights for the further investigation of USP45.

## Data Availability

The original contributions presented in the study are included in the article/Supplementary Material, further inquiries can be directed to the corresponding author.

## Supplementary Material

Supplementary Table S1 Prognostic Value of Single CpG of the USP45 gene in pan-cancer by MethSurv platform.

Supplementary Figure S1 Correlation analysis of USP45 with OS and RFS in patients with different types of tumors. The correlation of USP45 with OS in patients with BLCA **(A)**, EAC **(B)**, KIRC **(C)**, PCPG **(D)**, and RFS in patients with UCEC **(E)**, LIHC **(F)**, BRCA **(G)**, HNSC **(H)**, LUAD **(I)**, UCEC **(J)** were analyzed.

Supplementary Figure S2 Correlation analysis among USP45, miRNA with lncRNA in ceRNA network. In COAD **(A)** and PRAD **(B)**, the correlation among USP45, miR-30e-5p with AL137003.2 was analyzed. In KICH **(C)**, SARC **(D)**, LGG **(E)**, THCA **(F)** and PRAD **(G)**, the correlation among USP45, miR-425-5p with AL137003.2 were analyzed. In LGG **(H)**, the correlation among USP45, miR-328-3p with HELLPAR was analyzed.

Supplementary Figure S3 The construction of downstream regulatory network for USP45 in CHOL, NSCLC, MESO, SKCM.

Supplementary Figure S4 The construction of downstream regulatory network for USP45 in SARC, ESCA, UVM, ACC.

Supplementary Figure S5 The construction of downstream regulatory network for USP45 in LIHC, PRAD, RCC, HNSC, STAD.

Supplementary Figure S6 The construction of downstream regulatory network for USP45 in PAAD, UCS, UCEC, CESC, PCPG.

Supplementary Figure S7 The construction of downstream regulatory network for USP45 in BLCA, THCA, COADREAD, BRCA, GBMLGG.

Supplementary Figure S8 The correlation between the drugs sensitivity and USP45 in pan-cancer.
