# Supplementary figures and images for: Comprehensive Analysis Reveals USP45 as a Novel Putative Oncogene in Pan-Cancer
Source: Front Mol Biosci. 2022 Jun 28;9:886904. doi: 10.3389/fmolb.2022.886904 (PMC9273912; doi:10.3389/fmolb.2022.886904)

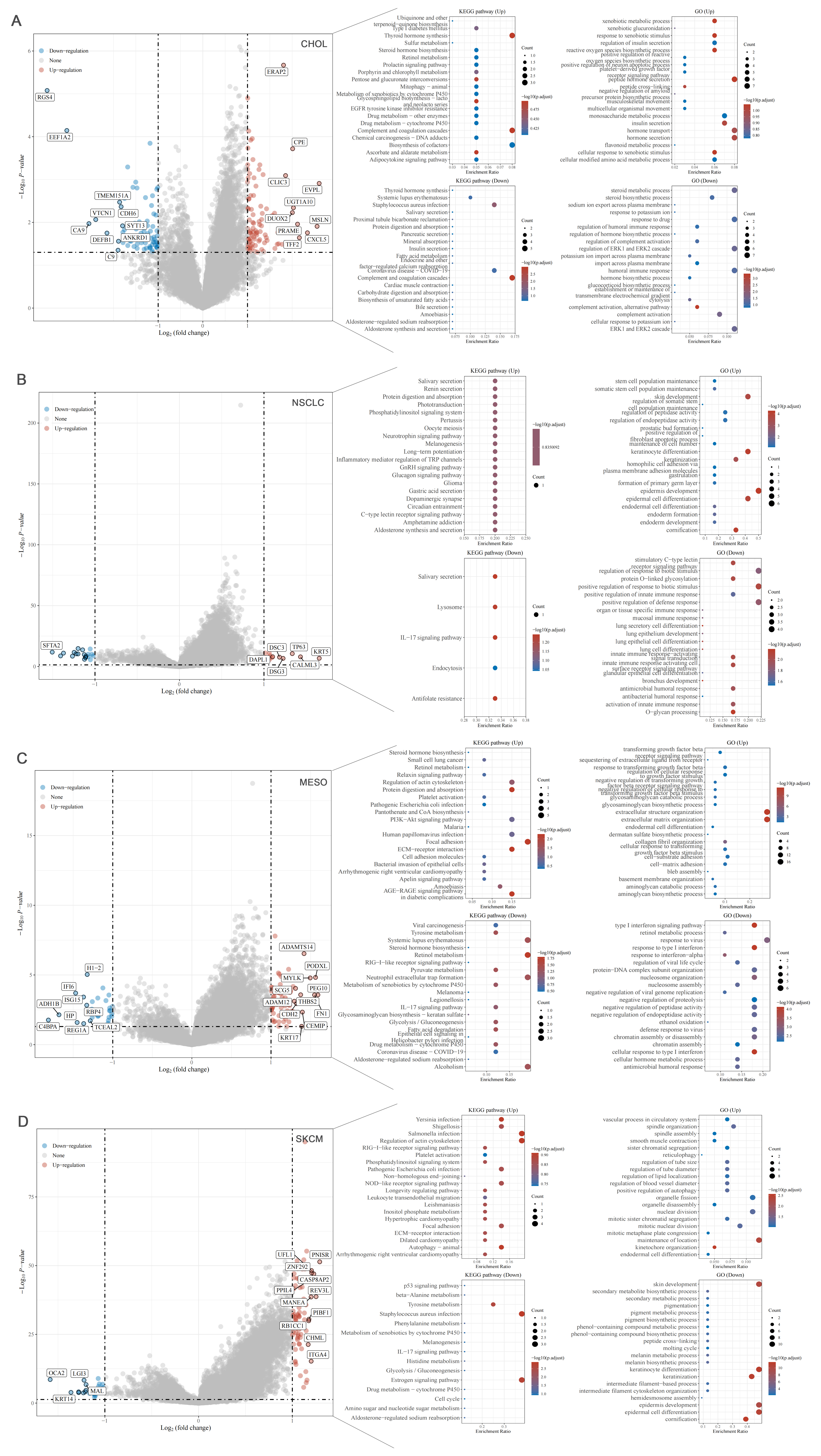

Supplement: Supplementary file 1 [file Image3.JPEG]

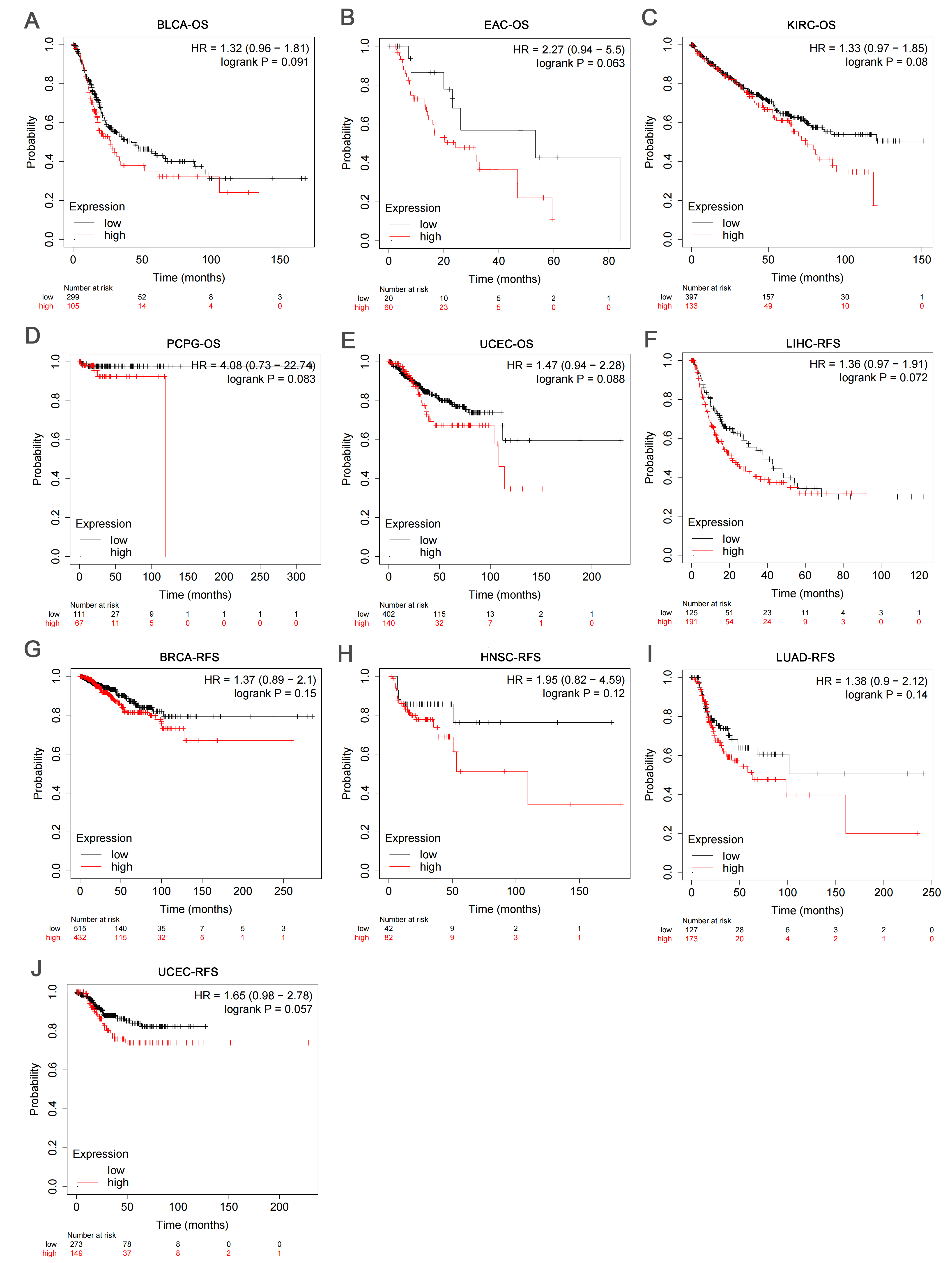

Supplement: Supplementary file 2 [file Image1.JPEG]

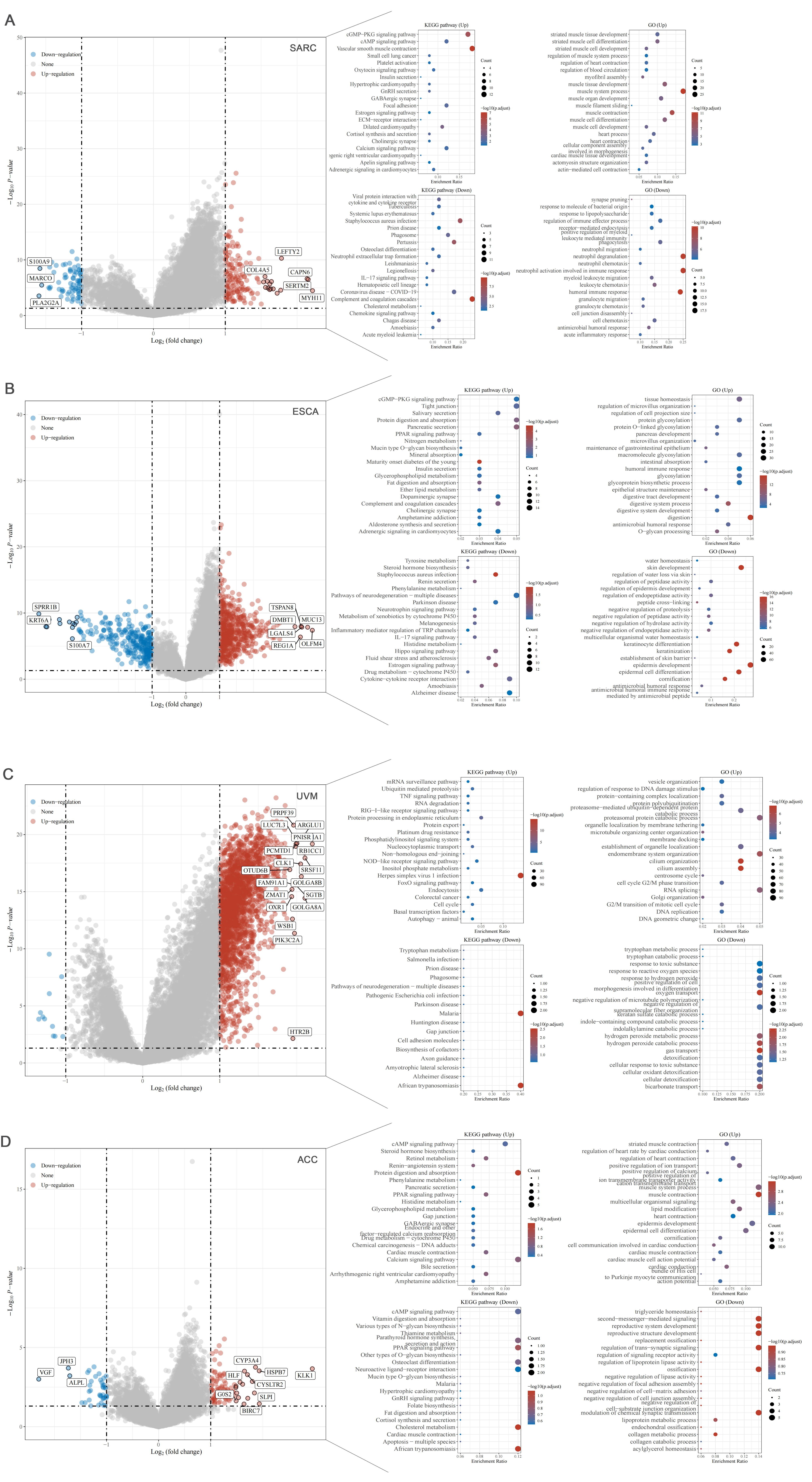

Supplement: Supplementary file 3 [file Image4.JPEG]

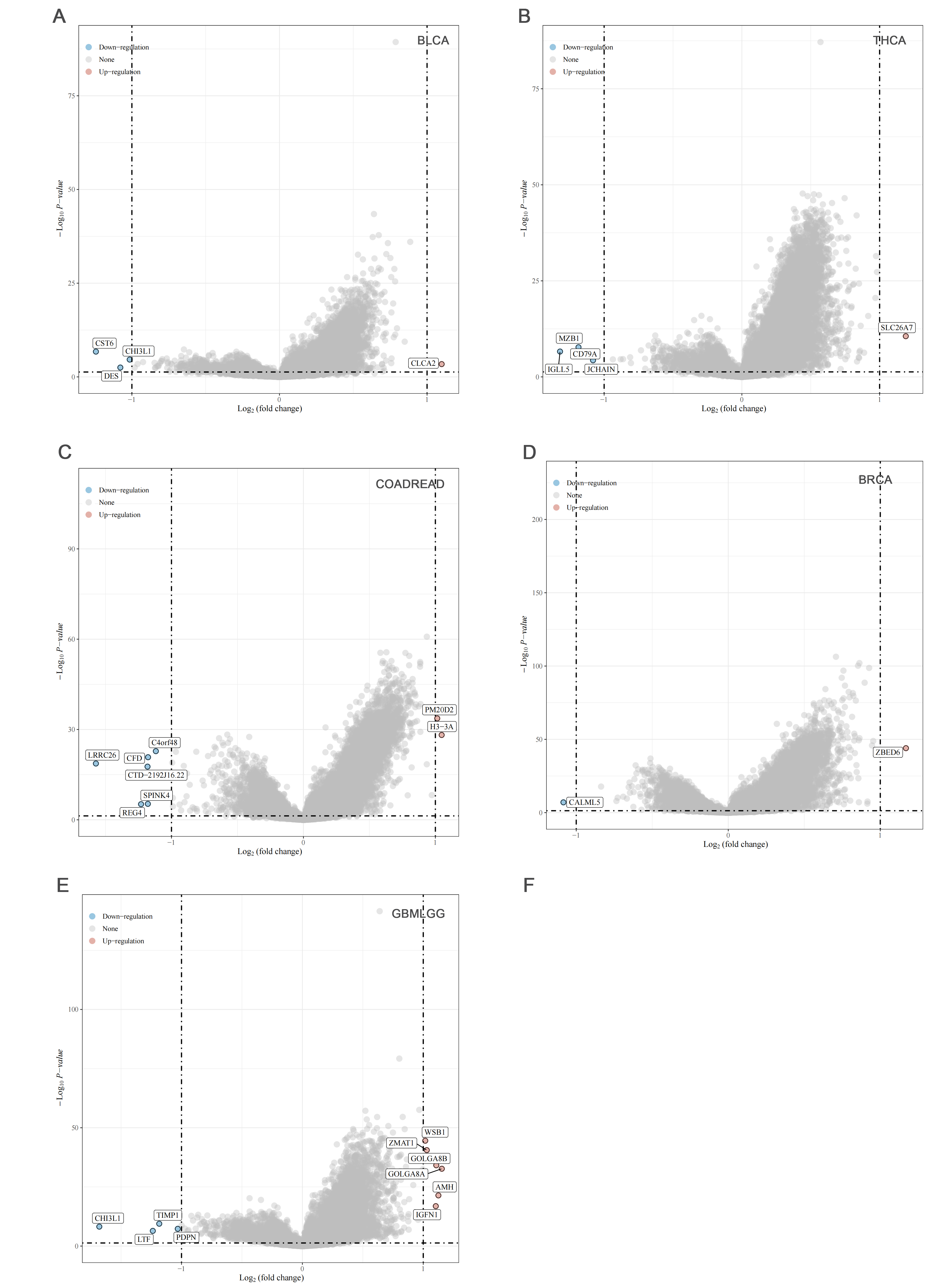

Supplement: Supplementary file 4 [file Image7.JPEG]

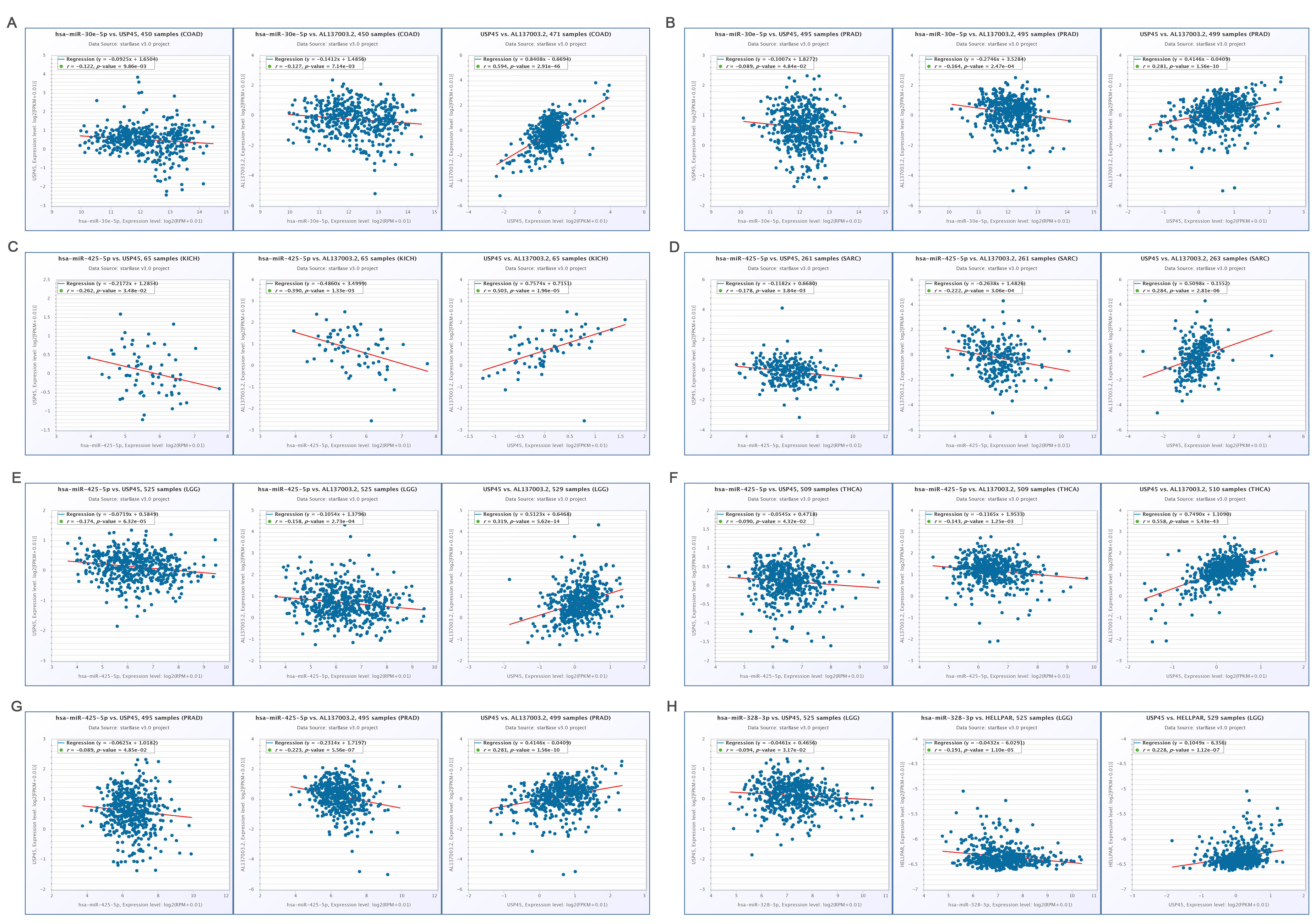

Supplement: Supplementary file 5 [file Image2.JPEG]

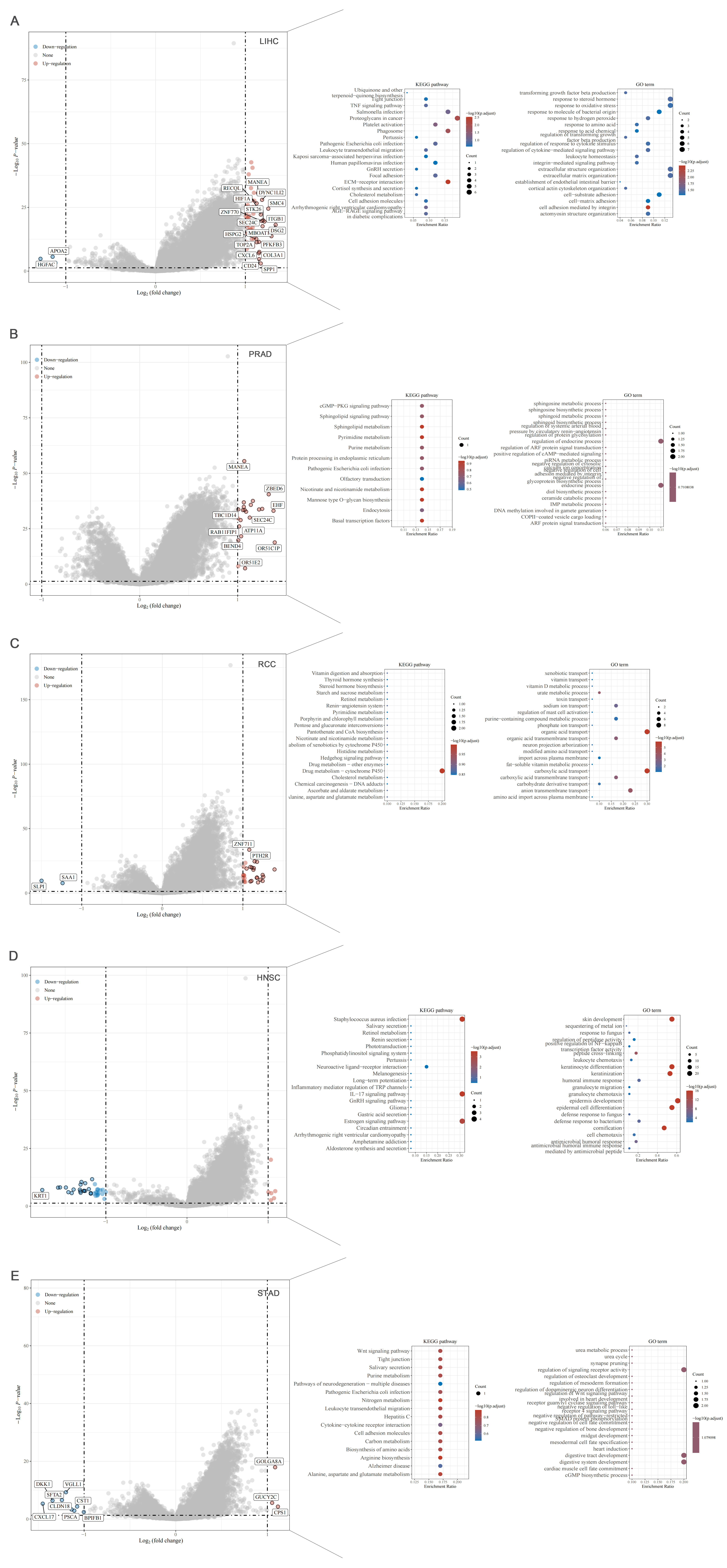

Supplement: Supplementary file 6 [file Image5.JPEG]

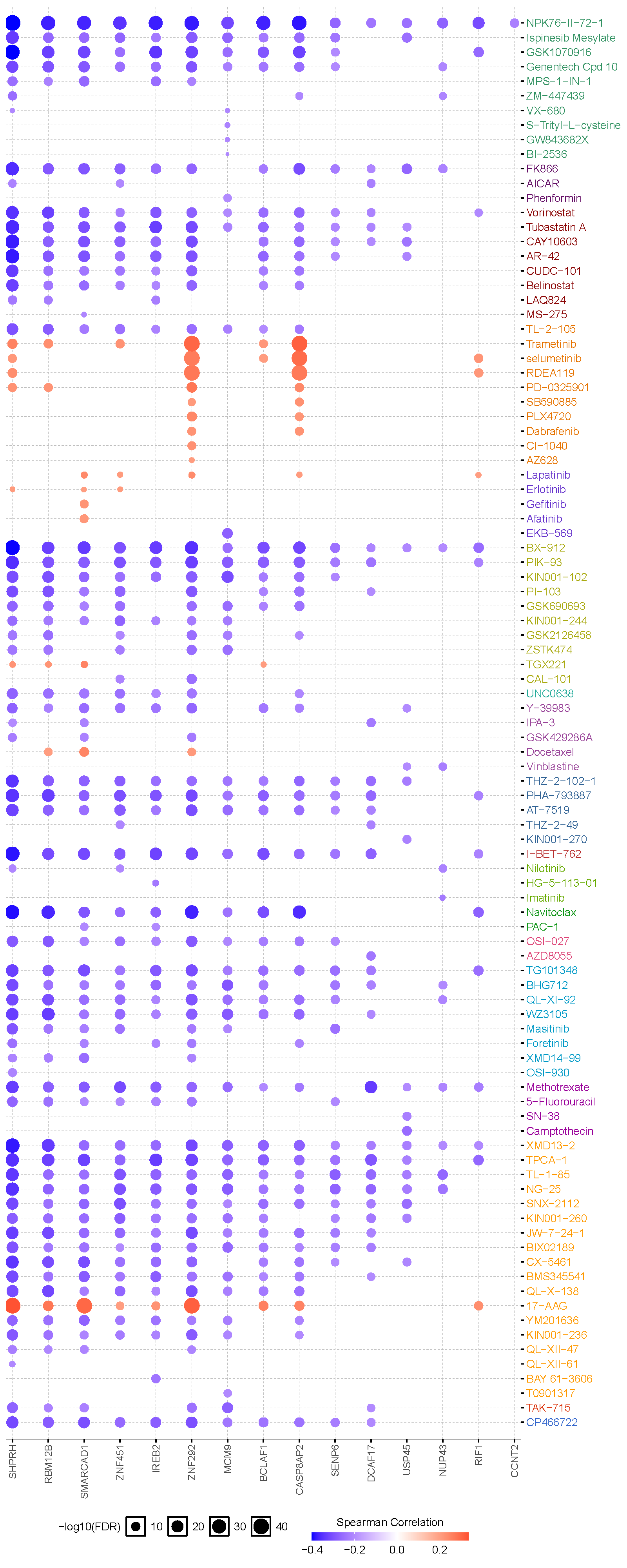

Supplement: Supplementary file 8 [file Image8.TIF]

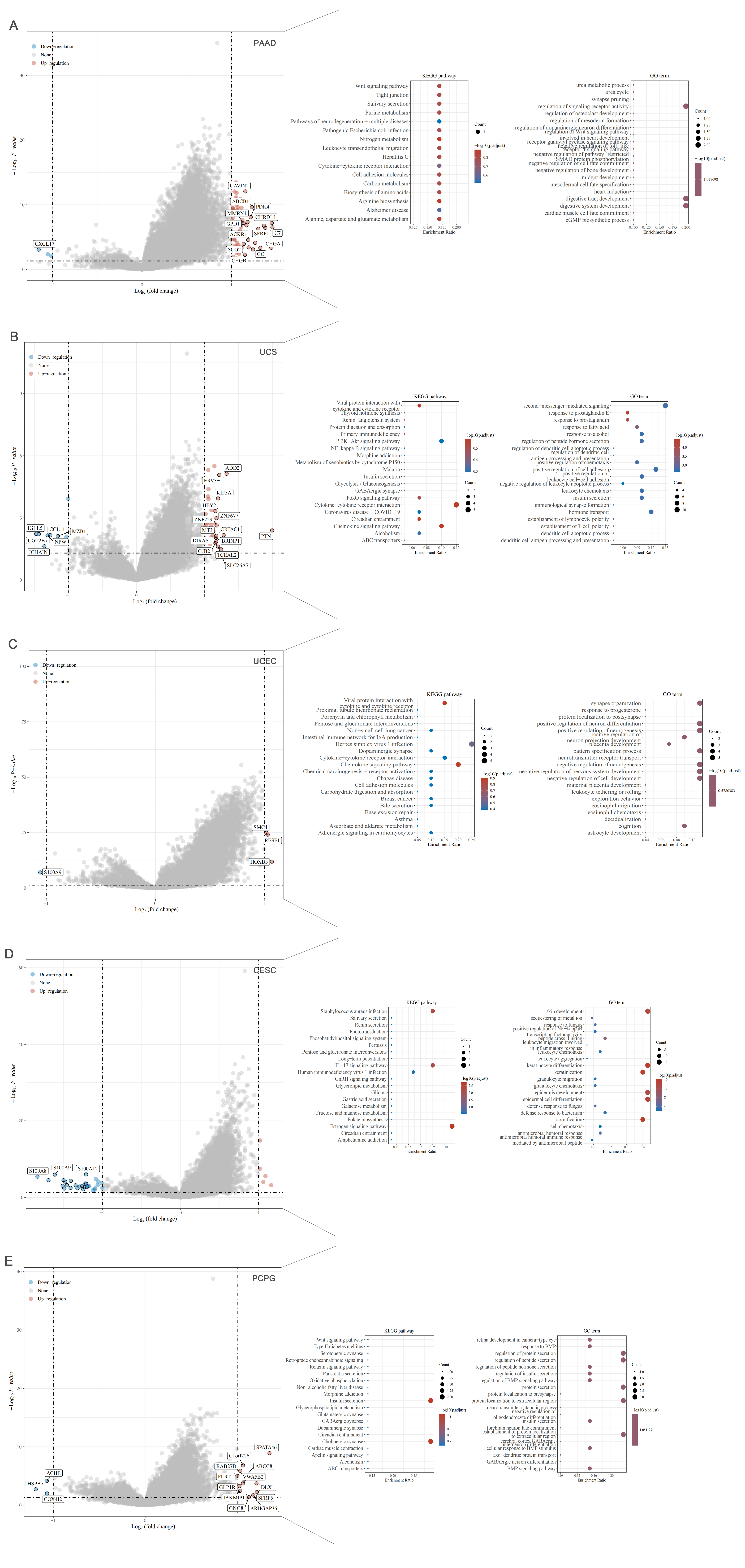

Supplement: Supplementary file 9 [file Image6.JPEG]
