# Supplementary material for: Comprehensive Analysis Reveals USP45 as a Novel Putative Oncogene in Pan-Cancer
Source: Front Mol Biosci. 2022 Jun 28;9:886904. doi: 10.3389/fmolb.2022.886904 (PMC9273912; doi:10.3389/fmolb.2022.886904)
Supplement: Supplementary file 7 [file Table1.doc]

| **Table S1. Prognostic Value of Single CpG of the USP45 gene in pan-cancer by MethSurv platform** | | | | |
| --- | --- | --- | --- | --- |
| **Cancer type** | **Gene Name** | **CpG** | **HR** | **LR_test_pvalue** |
| LAML | USP45 | 1stExon;5'UTR-  Island-  cg13363689 | 1.523 | 0.046707895 |
| LAML | USP45 | 3'UTR-  Open_Sea-  cg16486502 | 0.482 | 0.000771112 |
| ACC | USP45 | 3'UTR-  Open_Sea-  cg16486502 | 5.847 | 0.001947074 |
| BLCA | USP45 | 1stExon;5'UTR-  Island-  cg13363689 | 1.534 | 0.015457753 |
| BRCA | USP45 | 1stExon;5'UTR-  Island-  cg13363689 | 0.579 | 0.007147857 |
| BRCA | USP45 | TSS200-  Island-  cg13809693 | 1.602 | 0.040145943 |
| ESCA | USP45 | 1stExon;5'UTR-  Island-  cg13363689 | 0.44 | 0.000771131 |
| ESCA | USP45 | TSS200-  Island-  cg13809693 | 0.567 | 0.018330183 |
| ESCA | USP45 | TSS200-  Island-  cg17635724 | 0.594 | 0.025399844 |
| ESCA | USP45 | 5'UTR-  N_Shelf-  cg26484090 | 0.543 | 0.013323863 |
| GBM | USP45 | 1stExon;5'UTR-  Island-  cg08792129 | 0.648 | 0.041907141 |
| GBM | USP45 | 1stExon;5'UTR-  Island-  cg10139443 | 0.572 | 0.017089434 |
| GBM | USP45 | TSS1500-  S_Shore-  cg10970399 | 0.63 | 0.043624201 |
| GBM | USP45 | TSS1500-  Island-  cg25198784 | 0.59 | 0.02548444 |
| GBM | USP45 | TSS1500-  Island-  cg25309811 | 0.612 | 0.035882107 |
| GBM | USP45 | 5'UTR-  N_Shelf-  cg26484090 | 0.627 | 0.030298702 |
| KIRC | USP45 | 1stExon;5'UTR-  Island-  cg08792129 | 0.401 | 2.79E-05 |
| KIRC | USP45 | 1stExon;5'UTR-  Island-  cg10139443 | 0.484 | 0.004225693 |
| KIRC | USP45 | 1stExon;5'UTR-  Island-  cg13363689 | 0.561 | 0.01826678 |
| KIRC | USP45 | TSS200-  Island-  cg13809693 | 0.349 | 0.000166168 |
| KIRC | USP45 | TSS200-  Island-  cg17635724 | 0.43 | 0.000951197 |
| KIRC | USP45 | TSS1500-  Island-  cg25198784 | 0.453 | 0.00108245 |
| KIRC | USP45 | TSS1500-  Island-  cg25309811 | 0.546 | 0.004885722 |
| KIRC | USP45 | TSS1500-  Island-  cg27317327 | 0.407 | 0.000240439 |
| KIRP | USP45 | 1stExon;5'UTR-  Island-  cg21257892 | 2.918 | 0.019465466 |
| KIRP | USP45 | TSS1500-  Island-  cg25198784 | 2.291 | 0.01959156 |
| KIRP | USP45 | 5'UTR-  N_Shelf-  cg26484090 | 1.999 | 0.041481707 |
| KIRP | USP45 | TSS1500-  Island-  cg27317327 | 2.878 | 0.001194212 |
| LIHC | USP45 | TSS1500-  S_Shore-  cg10970399 | 1.598 | 0.010050384 |
| LIHC | USP45 | 5'UTR-  N_Shelf-  cg26484090 | 1.724 | 0.012400578 |
| LUAD | USP45 | 1stExon;5'UTR-  Island-  cg08792129 | 1.392 | 0.039631159 |
| LUAD | USP45 | TSS1500-  Island-  cg25198784 | 1.477 | 0.036674116 |
| MESO | USP45 | 1stExon;5'UTR-  Island-  cg21257892 | 1.737 | 0.024137019 |
| MESO | USP45 | TSS1500-  Island-  cg25198784 | 0.459 | 0.004789999 |
| MESO | USP45 | 5'UTR-  N_Shelf-  cg26484090 | 1.829 | 0.03098848 |
| PAAD | USP45 | 1stExon;5'UTR-  Island-  cg10139443 | 1.572 | 0.02920644 |
| READ | USP45 | 5'UTR-  N_Shelf-  cg26484090 | 3.047 | 0.027579364 |
| SARC | USP45 | TSS1500-  S_Shore-  cg10970399 | 0.559 | 0.00415361 |
| SARC | USP45 | TSS200-  Island-  cg17635724 | 0.633 | 0.039690775 |
| SARC | USP45 | TSS1500-  Island-  cg25309811 | 0.62 | 0.02099961 |
| SARC | USP45 | TSS1500-  Island-  cg27317327 | 0.514 | 0.002175513 |
| SKCM | USP45 | TSS1500-  S_Shore-  cg10970399 | 1.335 | 0.034077764 |
| STAD | USP45 | TSS1500-  Island-  cg25309811 | 0.68 | 0.03308546 |
| UCEC | USP45 | TSS1500-  S_Shore-  cg10970399 | 2.402 | 0.000212696 |
| UCEC | USP45 | TSS200-  Island-  cg13809693 | 1.679 | 0.028074958 |
| UCEC | USP45 | TSS1500-  Island-  cg25198784 | 2.491 | 0.000209743 |
| UCEC | USP45 | TSS1500-  Island-  cg25309811 | 2.009 | 0.017400562 |
| UCEC | USP45 | TSS1500-  Island-  cg27317327 | 1.94 | 0.004838825 |
| UCS | USP45 | TSS1500-  Island-  cg25198784 | 0.425 | 0.025204316 |
| UVM | USP45 | 5'UTR-  N_Shelf-  cg26484090 | 0.127 | 0.005070233 |
| The threshold of significance was LR Test p-value <0.05. A significant expression pattern was found in different CpG of USP45 between low and high risk groups for pan-cancer. | | | | |
